# Global analysis of the influence of environmental variables to explain ecological niches and realized thermal niche boundaries of sea snakes

**DOI:** 10.1371/journal.pone.0310456

**Published:** 2024-12-05

**Authors:** Carlos Patrón-Rivero, Luis Osorio-Olvera, Octavio Rojas-Soto, Xavier Chiappa-Carrara, Fabricio Villalobos, Brooke Bessesen, Kevin López-Reyes, Carlos Yañez-Arenas

**Affiliations:** 1 Laboratorio de Ecología Geográfica, Unidad de Conservación de la Biodiversidad, UMDI-Sisal, Facultad de Ciencias, Universidad Nacional Autónoma de Mexico, Sierra Papacal, Yucatán, Mexico; 2 Laboratorio de Ecoinformática de la Biodiversidad, Departamento de Ecología de la Biodiversidad, Instituto de Ecología, Universidad Nacional Autónoma de México, Circuito Exterior s/n Anexo al Jardín Botánico, Ciudad Universitaria, Coyoacán, Ciudad de México, México; 3 Laboratorio de Bioclimatología, Red de Biología Evolutiva, Instituto de Ecología A.C., Xalapa, Veracruz, México; 4 Departamento de Sistemas y Procesos Naturales, Escuela Nacional de Estudios Superiores Unidad Mérida, Universidad Nacional Autónoma de Mexico, Ucú, Yucatán, Mexico; 5 Laboratorio de Macroecología Evolutiva Red de Biología Evolutiva, Instituto de Ecología, A.C, Xalapa, Veracruz, México; 6 Department of Ecology and Evolutionary Biology, University of Reading, Reading, United Kingdom; Laboratoire de Biologie du Développement de Villefranche-sur-Mer, FRANCE

## Abstract

Understanding the factors affecting species distributions is a central topic in ecology and biogeography. However, most research on this topic has focused on species inhabiting terrestrial environments. At broad scales, abiotic variables consistently serve as primary determinants of species’ distributions. In this study, we investigated the explanatory power of different abiotic variables in determining the distribution patterns of sea snakes on a global scale. Additionally, as the boundaries of realized thermal niches have significant implications for the ecology of species and their geographic distributions, we evaluated the asymmetry of realized thermal limits (i.e., differences in variances between the upper and lower limits of the realized thermal niche). We obtained 10 marine environmental variables from global databases along with >5000 occurrence records for 51 sea snake species in 4 genera across the group’s entire known geographic range. Using these data, we employed correlative ecological niche modeling to analyze the influence of the individual variables in explaining species’ distributions. To estimate the realized thermal limits of each species, we extracted the mean, minimum, and maximum temperature values at four depths (superficial, mean benthic, minimum benthic, and maximum benthic) for each occurrence record of the species. We then evaluated the asymmetry of the realized thermal niche by measuring and comparing the variances in the upper and lower limits. Both analyses (the importance of variables and realized thermal limit asymmetry) were performed at three taxonomic levels (sea snakes as a lineage of marine-adapted elapids [true sea snakes + sea kraits], subfamily, and genus) and two spatial resolutions. Overall, we found that temperature, silicate, nitrate, salinity, and phosphate concentrations were the most influential factors in explaining the spatial distribution patterns of sea snakes, regardless of taxonomic level or spatial resolution. Similarly, we observed that the realized thermal limits were asymmetric, with a higher variance in the lower limits, and that asymmetry decreased as the taxonomic level and spatial resolution increased.

## Introduction

Anthropogenic activities have led to rapid global changes that create unprecedented climatic conditions, which could compromise the viability of many species [1, 2]. Understanding how environmental conditions affect the survival of species’ populations and shape their distributions is fundamental for establishing conservation strategies [3]. However, estimating the relationships between species and their environments is a complex task, as it involves multiple factors [4–6]. These factors can be summarized into three categories: 1) limiting or regulatory factors, which control the ecophysiological responses of the species, 2) disturbance factors, which describe the historical modifications in the occupation of habitats by both natural and anthropogenic processes, and 3) resource factors, which represent the supplies necessary for the survival of organisms [7]. Some causal relationships between these factors and species’ attributes, such as abundance and distribution, have been demonstrated through physiological experiments in laboratories and wildlife demographic studies [8–10]. Conducting laboratory experiments or fieldwork to obtain demographic data has certain drawbacks, however. First, both are generally costly and time-consuming. Second, implementing physiological experiments is not ethically feasible for many organisms [11]. Finally, some environmental variables are found to be more relevant at broad spatial scales (>200 km); for instance, limiting or regulating factors associated with climate are considered most relevant in explaining the distribution of species across their entire range [12–14].

An alternative approach to studying species-environment relationships in a spatial context is the use of correlative methods, known generically as ecological niche modeling (ENM) and species distribution modeling (SDM) [14–16]. The inputs required by correlative methods are easier to acquire, because they are generally available in online repositories. These inputs are sets of georeferenced localities where the presence of the species in question has been observed (some methods also require points that represent absence), and spatial surfaces that describe the environmental conditions of the area of interest [17]. Thus, correlative methods are extensively used to model species-environment relationships and estimate their actual and potential distributions, both in terrestrial [18, 19] and marine ecosystems [3, 20]. Although correlative methods have certain limitations [e.g., it is difficult to discern whether their results are merely statistical associations or causal relationships; see 21], they allow us to recognize diverse ecological patterns [22]. As a result, these methods have become popular for analyzing the importance of environmental variables as limiting factors for distribution ranges and various ecological aspects across multiple taxa [3, 20, 23].

One of the most studied limiting factors is temperature, which is important for many physiological and ecological processes [10, 24, 25]. Thus, studying the thermal niche (TN) and realized thermal niche (RTN) of species is a major focus of ecological research [23, 26–28]. According to Gvoždík [26], TN refers to the range in body temperature that individuals of a species require for the population to experience positive growth, whereas RTN refers to the environmental range of temperatures to which individuals of a species are exposed [28, 29]. Based on the concepts of TN and RTN, two hypotheses have been proposed to explain the patterns of temperature tolerance and organismal response to global warming. The first hypothesis suggests that tropical and polar terrestrial species have lower TN than those from temperate climates [30, 31]. The second hypothesis is realized thermal limit asymmetry, which refers to significant differences in the variation of upper limits in a taxon compared with the variation of lower limits within the same taxon, with the variation being greater in the lower limits. Lower variances in upper thermal limits have been reported to severely impact marine species, which may face increasing sea temperatures under climate change scenarios with the potential to impact both survivability and habitat suitability [28, 29, 32–34]. Ideally, evaluating these hypotheses requires direct estimation of the species’ TN through physiological population growth-mortality experiments [35–38], and so despite the high cost, time and ethical constraints, there continue to be global initiatives to analyze those patterns [39]. Unsurprisingly, the characterization of the TNs for most taxa is still lacking.

Because correlative methods do not require experimentation and management of organisms, they are a more feasible option for studying species’ TNs. Consequently, they have been commonly applied to many taxonomic groups [37, 40–42]. Most TN analyses have focused on species from terrestrial environments [39], though using correlative methods, it is possible to characterize the RTN as the range of temperatures associated with the presence of a species [43–45], allowing investigations of many important marine groups, such as sea snakes.

Sea snakes are an ecologically diverse and marine-adapted lineage of elapids that includes two subfamilies: Hydrophiinae (true sea snakes) and Laticaudinae (sea kraits) [46–48]. This group comprises >70 species [49] that are widely distributed in the tropical and subtropical regions of the Indian and Pacific Oceans [50, 51]. Sea snake lineages have evolved unique physiological adaptations to survive in marine environments [52, 53]. Research has shown temperature to play a critical role in their distribution [54–56]. Moreover, individuals exhibit different diving behaviors and metabolic rates in response to changes in water temperature [57, 58]. These findings highlight the complex ways in which temperature influences the ecology and physiology of sea snakes; yet, to date, there is limited information on the asymmetry of their realized thermal limits or the broader environmental factors that influence their distribution. In this study, we used correlative ecological niche modeling (ENM) to evaluate the relative importance of environmental variables in explaining the distribution of a majority of sea snake species globally. We also analyzed whether there was asymmetry in the dispersion of the lower and upper realized thermal limits. We underscore the importance of understanding the thermal biology of these reptiles for conservation and management efforts. The results of our research provide a better understanding of the biogeographical phenomena that determine the distribution patterns in this group and will allow us to infer their vulnerability to future ocean warming scenarios.

## Materials and methods

### Presence records

We searched the presence records with geographic coordinates for all sea snakes in the public online repositories following the available phylogenies and agreements [49, 59–61]. These platforms are the Global Biodiversity Information Facility (gbif.org; DOI information associated with data are available in S1 Table), VertNet (vertnet.org), the Ocean Biogeographic Information System (obis.org), the Atlas of Living Australia (ala.org.au), the Online Zoological Collections of Australian Museums (ozcam.org.au) and the UNAM open data portal (datosabiertos.unam.mx). We integrated the presence records obtained from the different platforms into a single database for each species. We then cleaned the data by eliminating duplicate records, uncertain data (e.g., taxonomic mistakes, records outside the known distribution of the group), and obvious errors (e.g., records in terrestrial environments). Next, we eliminated occurrences that might represent sink populations through an environmental outliers’ analysis. In this process, we considered the environmental variables previously identified in the literature as important to the group (see below), using the *outliers* function of the “biogeo” package [62] in R [63], which searches for presence records that are located at a distance above 1.5 times the interquartile range of the data. Additionally, we employed the *gridSample* function of the “dismo” package [64] to minimize data bias towards more intensely sampled environments. Finally, we split the data that include historical and other with only contemporary records for constructing the **M** area and for the modeling process, respectively (see below). These processed records established a study database of >5000 usable presence records comprising a total of 51 species in four genera: *Aipysurus*, *Emydocephalus*, *Hydrophis*, and *Laticauda*, with record counts by species ranging from 5–788 (x̄ = 100; Table 2).

### Environmental data

From the global repositories Bio-Oracle [65] and MARSPEC [66], we downloaded raster formats of environmental variables commonly used in spatial modeling of marine environments: calcite (Cal), current velocity (Cvel), dissolved molecular oxygen (Doxy), iron (Iro), nitrate (Nit), pH, phosphate (Pho), salinity (Sal), silicate (Sil), and temperature (Tem) obtained at four depths (superficial, benthic maximum, benthic mean, and benthic minimum). We then classified the variables into five groups: Bsurf (superficial Bio-Oracle; n = 10), Bma (maximum benthic Bio-Oracle; n = 8), Bme (mean benthic Bio-Oracle; n = 8), Bmi (minimum benthic Bio-Oracle n = 8), and Msurf (superficial MARSPEC; n = 6). This protocol was used to select the combination of repository/depth that maximized the explanatory capacity of the predictors (Table 1). To standardize the spatial resolution of the variables, we resampled the MARSPEC variables to match the 5 arc-minutes (arcmin) cell resolution of Bio-Oracle. Subsequently, we resampled both repositories at a resolution of 10 arcmin using the same method to assess the effect of spatial resolution on our results [67].

**Table 1:** Variable groups with description of each individual variable by repository and depth (in parenthesis).

Variable	VCode	Description of variable
Calcite	Cal	Surface calcite (Bsuf)
Currents velocity	Cvel	Mean surface currents velocity (Bsuf)
		Mean benthic max currents velocity (Bma)
		Mean benthic mean currents velocity (Bme)
		Mean benthic min currents velocity (Bmi)
Dissolved molecular oxygen	Doxy	Mean surface dissolved molecular oxygen (Bsuf)
		Mean benthic max dissolved molecular oxygen (Bma)
		Mean benthic mean dissolved molecular oxygen (Bme)
		Mean benthic min dissolved molecular oxygen (Bmi)
Iron	Iro	Mean surface iron (Bsuf)
		Mean benthic max iron (Bma)
		Mean benthic mean iron (Bme)
		Mean benthic min iron (Bmi)
Nitrate	Nit	Mean surface nitrate (Bsuf)
		Mean benthic max nitrate (Bma)
		Mean benthic mean nitrate (Bme)
		Mean benthic min nitrate (Bmi)
pH	pH	Surface pH (Bsuf)
Phosphate	Pho	Mean surface phosphate (Bsuf)
		Mean benthic max phosphate (Bma)
		Mean benthic mean phosphate (Bme)
		Mean benthic min phosphate (Bmi)
Salinity	Sal	Mean surface salinity (Bsuf)
		Mean benthic max salinity (Bma)
		Mean benthic mean salinity (Bme)
		Mean benthic min salinity (Bmi)
		Mean annual sea surface salinity (Msurf)
		Sea surface salinity of the freshest month (Msurf)
		Sea surface salinity of the saltiest month (Msurf)
Silicate	Sil	Mean surface silicate (Bsuf)
		Mean benthic max silicate (Bma)
		Mean benthic mean silicate (Bme)
		Mean benthic min silicate (Bmi)
Temperature	Tem	Mean surface temperature (Bsuf)
		Mean benthic max temperature (Bma)
		Mean benthic mean temperature (Bme)
		Mean benthic min temperature (Bmi)
		Mean annual sea surface temperature (Msurf)
		Sea surface temperature of the coldest month (Msurf)
		Sea surface temperature of the warmest month (Msurf)

Notes: VCode = variable code. Bma = Bio-Oracle benthic maximum, Bme = Bio-Oracle benthic mean, Bmi = Bio-Oracle benthic minimum, Bsurf = Bio-Oracle surface, Msurf = MARSPEC surface.

For the study range of each species, we defined a polygon representing a hypothesis of historically accessible areas [area **M**; *sensu* 68] based on the marine biogeographical provinces of the world [69]. We first selected all provinces with at least one presence record of the species in question and then we took species dispersal into account, using ocean currents information (earth.nullschool.net/#current/ocean/surface/currents/patterson) [70]. If the **M** area appeared fragmented into “provinces,” we systematically evaluated the likelihood of those provinces being connected. For provinces containing numerous and also widely distributed presence records, we hypothesized that ocean currents could serve as dispersal channels creating connectivity between them [71]. In this case, we added provinces to form a unified area. Meanwhile, for species with unlinked provinces showing only isolated records, we considered currents as potential physical barriers and removed any provinces between. We also defined a calibration area and masked the variables by selecting only the areas within the intersection between the presence records and the marine eco-regions, a finer sub-regionalization than the provinces. Finally, to reduce collinearity and dimensionality of predictors, we eliminated individually for each species, variables in each of the five groups that had a pairwise Pearson correlation >0.8 with the function *correlation_finder* in “ntbox” package [72–74].

### Ecological niche models

To characterize the ecological niches of sea snakes, we used the MaxEnt 3.4.1 algorithm [75]. We used the jackknife cross-validation procedure to improve the accuracy and minimize the variability of the models in taxa with 5–25 localities. In this method, one presence record is excluded randomly from the modeling, and the procedure is repeated as many times as the data allow; all records are excluded just once. In each iteration, *n*-1 data were used as model training information, and the excluded record was used for model evaluation [76]. Finally, for species with more than 25 occurrence records, we applied the *get*.*checkerboard1* function in the “ENMeval” package [77]. In this method, the database was divided into two sets using a checkerboard pattern across the study area. Occurrences were separated according to their position on the board. We defined the set with more records as training data and the set with less information as the evaluation data [78].

A series of candidate models were calibrated with the training database for each species via the “kuenm” package [79]. This package allows for varying the parameter combinations of the MaxEnt algorithm. We tested combinations of seven feature classes in MaxEnt (“l”, “q”, “p”, “lq”, “lqp”, “lp”, and “qp”, where “l” is linear, “q” is quadratic, and "p" is product), eight regularization multipliers (0.10, 0.25, 0.50, 0.75, 1, 2, 3 and 4), and the five groups of environmental variables described above (thus, the maximum possible number of variables used to build any model is full set of “repository/depth” if no correlation between all variables were found). This resulted in 560 candidate models per species (spp × 2 resolutions × 7 Fs × 8 RMs × 5 groups of variables). Then we selected using the test database the best subset of models that met the following criteria hierarchically: 1) statistical significance, noting that due to limitations in SDMs and ENMs related to the sensitivity of the area used in model construction, we did not rely on the traditional AUC for model evaluation; instead, we utilized the partial AUC ratio, which penalizes the quantity of area involved in the estimation and is a more informative metric, widely accepted in the literature [80]; we retained those models that were better than expected by chance, depending on the proportion of bootstrap replicates with ratios of the partial area >1 [81]; 2) predictive capacity, where we selected models that were also able to predict at least 90% of the evaluation records (i.e., models with an omission rate–OR–≤ 0.10); and 3) complexity, by using Akaike information criterion corrected for small samples (ΔAICc) we selected as best combinations those ≤ 2 units, that are also the models that present a better fit and fewer parameters [82, 83]. Based on the best parameter combinations (full set of best combinations are available in S2 Table), we built a final set of models using the entire database (training and evaluation data) with the bootstrap functionality of MaxEnt performing 10 replicates. In each iteration, we randomly divided the presence records into 80% for training and 20% for evaluation, and defined Cloglog as output format with 10,000 background points (masked by our calibration area). Finally, we calculated the median and range of the predicted values across the total replicates (10 replicates × final parameter combination) to represent the consistency and variation in the predictions.

### Importance of variables

To analyze the relative importance of environmental variables in explaining sea snakes’ distributions, we first classified the variables into ten groups based on their identity. For example, the group “Temperature (Tem)” included seven variables that represented the median of the mean, maximum and minimum values of the sea surface and benthic temperature (Table 1).

We used two measures to assess the relative importance of the environmental variables as estimated by MaxEnt: percentage of contribution (PC) and permutation of importance (PI). PC measures the gain of a model built with a single specific variable divided by the total gain of a model built with all variables. PI is obtained by randomizing the values of each variable so that this variable is not informative and then measuring the resulting drop in the area under the curve [84]. To identify the consistency or variability in our results, we performed this procedure at three levels: “lineage”, where we analyzed the variables for all species regardless of subfamily; “subfamily”, where we divided the results into the subfamilies Hydrophiinae and Laticaudinae [46–48]; and “genus”, where we repeated the same analysis for each of the four genera. We performed this analysis at both resolutions (5 and 10 arcmin). We also separated the identity variables into two groups (high relative importance and low relative importance) based on their relative importance using the natural Jenks thresholds calculated with the *getJenksBreaks* function of the “BAMMtools” package [85].

To determine if the results depended on spatial resolution, we evaluated the consistency of the importance of each variable between the two spatial resolutions for the two MaxEnt metrics (PC and PI) through a non-parametric Mann-Whitney-Wilcoxon test using the *wilxicon*.*test* function in “stats” package [63]. Overall, this approach allowed us to identify the most important environmental variables driving the distribution of sea snakes and to assess the consistency of these results across different metrics of importance, taxonomic levels, and spatial resolutions.

### Asymmetry of the realized thermal niche (RTN)

To assess asymmetry in the RTNs, we extracted the mean, maximum, and minimum temperature values for the five groups depending on the repository and depth (Bsurf, Bma, Bme, Bmi, and Msurf) associated with each post processed presence record (see above in “2.1 Presence records”**)** of each species at 5 and 10 arcmin of spatial resolution. For Bio-Oracle, we directly used the mean, maximum, and minimum temperature variables at each depth. For MARSPEC, we used the surface temperature of the warmest month to represent the maximum temperature and the surface temperature of the coldest month to represent the minimum temperature. Using these values, we conserved and grouped the minimum and maximum values from each species to characterize the lower and upper RTN limits respectively.

Finally, we tested the asymmetry in the RTN limits hypothesis (asymmetry in the variances with more variance in the lower limit) by testing the homogeneity of the variances between the lower and upper realized thermal limits. This process was repeated for each variable group at each temperature measurement at the three taxonomic levels and two spatial resolutions by applying a non-parametric Fligner-Killeen test [86] (5 groups × 3 temperatures × 3 taxonomic levels × 2 resolutions). Importantly, at the genus level, we excluded *Laticauda* as it contains the identical species as the Laticaudinae subfamily.

## Results

Of our 5,023 spatially unique presence records for 51 of the >70 recognized sea snake species [49, 60, 87], most were distributed in the Indo-Pacific Ocean, with *Hydrophis platurus* being the only species found in the eastern Pacific Ocean of the New World.

Regarding model evaluation, we obtained a median partial AUC ratio >1 for all species (i.e., all our models were better than expected by chance). The ORs in the final models were ≤ 0.10, with the exception of three species when using the variables at 5 arcmin resolution and six at 10 arcmin (Table 2).

**Table 2:** Summary of inputs and evaluations from ecological niche modeling.

Spp	To	Fo	M5	M10	Ar5	Ar10	Or5	Or10
*Aipysurus apraefrontalis*	22	14	7	4	1.87	1.88	0.00	0.00
*Aipysurus dubosii*	287	128	5	1	1.71	1.67	0.10	0.10
*Aipysurus eyddouxii*	452	205	1	1	1.80	1.88	0.08	0.09
*Aipysurus foliosquamata*	17	8	12	1	1.83	1.86	0.00	0.00
*Aipysurus fuscus*	33	14	2	4	1.99	1.91	0.00	0.00
*Aipysurus laevis*	1281	385	1	2	1.75	1.37	0.09	0.09
*Aipysurus mosaicus*	145	61	1	2	1.74	1.65	0.09	0.09
*Aipysurus pooleorum*	18	7	10	2	1.94	1.70	0.00	0.00
*Aipysurus tenuis*	16	5	24	33	1.68	1.69	0.00	0.00
*Emydocephalus annulatus*	115	54	1	3	1.72	1.74	0.07	0.07
*Emydocephalu ijimae*	11	8	16	3	1.99	1.84	0.00	0.00
*Hydrophis atriceps*	45	24	8	17	1.79	1.77	0.00	0.00
*Hydrophis belcheri*	17	11	29	1	1.78	1.74	0.00	0.00
*Hydrophis brookii*	15	9	1	4	1.88	1.96	0.00	0.00
*Hydrophis caerulescens*	48	30	3	6	1.59	1.56	0.06	0.06
*Hydrophis coggeri*	33	16	8	5	1.74	1.39	0.00	0.00
*Hydrophis curtus*	339	133	1	1	1.71	1.45	0.10	**0.11**
*Hydrophis cyanocinctus*	86	45	8	7	1.60	1.22	0.09	0.09
*Hydrophis czeblukovi*	16	8	6	23	1.95	1.96	0.00	0.00
*Hydrophis elegans*	3142	788	1	1	1.89	1.89	0.10	0.10
*Hydrophis fasciatus*	35	23	10	6	1.71	1.74	0.00	0.00
*Hydrophis gracilis*	34	19	10	6	1.65	1.76	0.00	0.00
*Hydrophis hardwickii*	1236	512	1	2	1.42	1.44	0.09	0.10
*Hydrophis inornatus*	14	6	21	28	1.84	1.81	0.00	0.00
*Hydrophis jerdonii*	5	5	3	1	1.64	1.79	0.00	0.00
*Hydrophis kingii*	197	121	4	1	1.57	1.42	0.08	**0.11**
*Hydrophis klossi*	8	5	2	6	1.99	1.99	0.00	0.00
*Hydrophis lapemoides*	30	18	1	1	1.88	1.90	0.00	0.00
*Hydrophis macdowelli*	100	62	2	2	1.44	1.44	0.07	0.07
*Hydrophis major*	1099	404	1	1	1.91	1.92	**0.14**	**0.11**
*Hydrophis melanocephalus*	23	14	1	1	1.98	1.99	0.00	0.00
*Hydrophis melanosoma*	26	18	6	9	1.30	1.31	**0.20**	**0.20**
*Hydrophis nigrocinctus*	12	6	6	6	1.92	1.92	0.00	0.00
*Hydrophis ocellatus*	251	113	1	1	1.4	1.56	0.10	0.10
*Hydrophis ornatus*	870	347	1	1	1.69	1.67	0.09	**0.11**
*Hydrophis pacificus*	251	108	1	1	1.61	1.64	0.09	0.09
*Hydrophis peronii*	325	170	1	1	1.66	1.41	0.09	**0.12**
*Hydrophis platurus*	935	403	1	1	1.67	1.72	0.09	0.08
*Hydrophis schistosus*	22	11	1	3	1.55	1.17	0.00	0.00
*Hydrophis spiralis*	21	14	13	8	1.71	1.84	0.00	0.00
*Hydrophis stokesii*	666	300	1	1	1.83	1.82	0.09	0.10
*Hydrophis torquatus*	14	6	3	18	1.99	1.99	0.00	0.00
*Hydrophis viperinus*	26	16	8	8	1.93	1.87	0.00	0.00
*Hydrophis zweifeli*	17	9	12	10	1.52	1.43	0.00	0.00
*Laticauda colubrina*	419	219	1	1	1.84	1.36	0.08	0.10
*Laticauda crokeri*	12	5	1	12	1.69	1.98	0.00	0.00
*Laticauda frontalis*	10	7	20	16	1.97	1.98	0.00	0.00
*Laticauda laticaudata*	112	71	2	3	1.70	1.60	0.07	0.09
*Laticauda saintgironsi*	59	32	3	8	1.68	1.60	**0.11**	0.06
*Laticauda schistorhyncha*	17	7	3	1	1.99	1.99	0.00	0.00
*Laticauda semifasciata*	31	19	1	3	1.41	1.88	0.00	0.00

Notes: To = total occurrences, Fo = final post-processed occurrences, M5 = number of final models at 5 arc-minutes, M10 = number of final models at 10 arc-minutes, Ar5 = median of AUC ratios at 5 arc-minutes, Ar10 = median of AUC ratios at 10 arc-minutes, Or5 = median of omission rate at 5 arc-minutes, Or10 = median of omission rate at 5 arc-minutes, bold = omission rate > 10%. The subfamily Laticaudinae consists only of the genus Laticauda, whereas the remaining genera form the subfamily Hydrophiinae.

### Importance of variables

**Fig 1:**
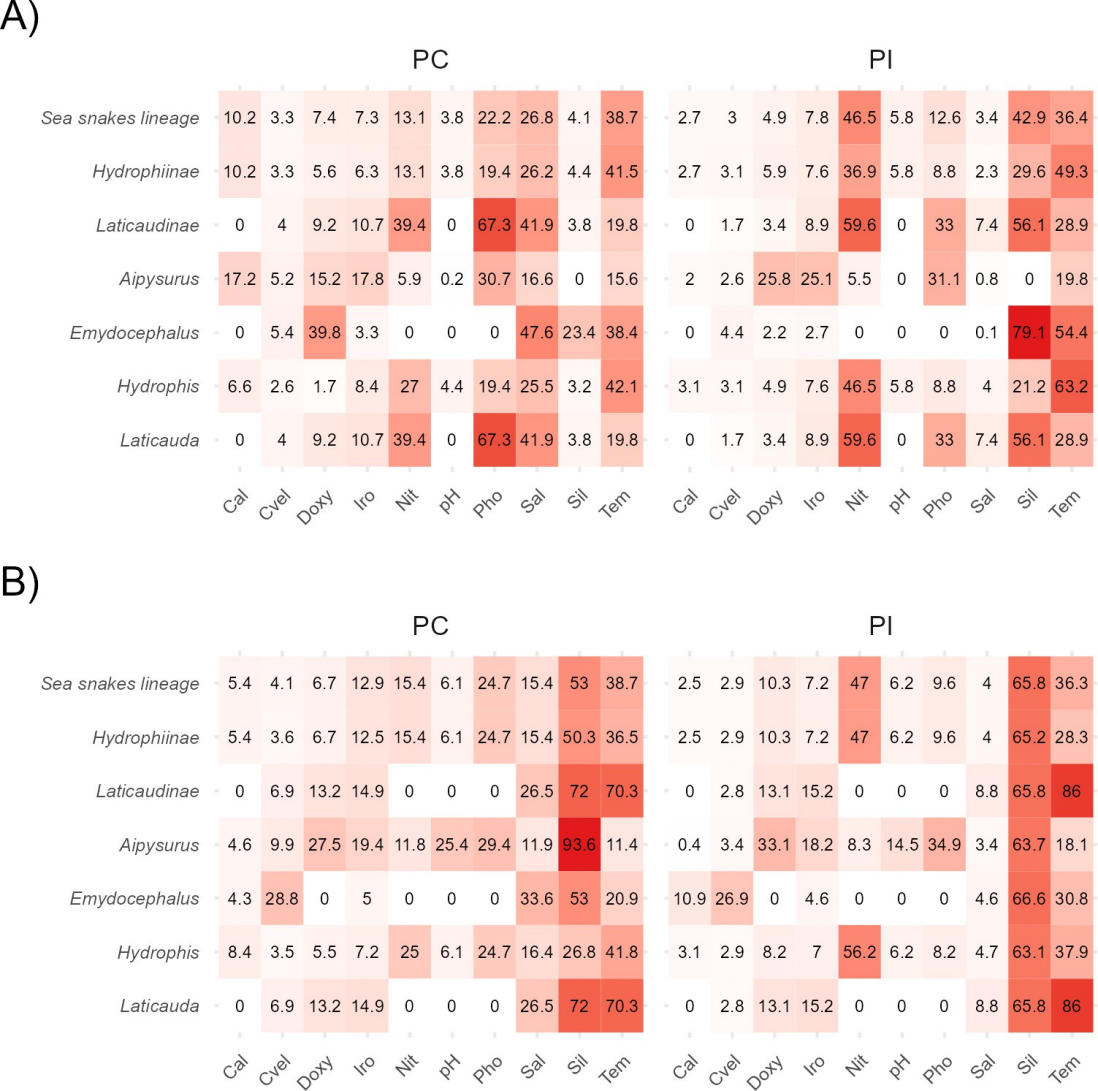
Variable contribution estimated by MaxEnt percentage of contribution (PC) and permutation of importance (PI) for each taxonomic level at both spatial resolutions. A) = 5 arcmin, B) = 10 arcmin. Darker colors denoted higher contribution while lighter colors less contribution.

**Fig 2:**
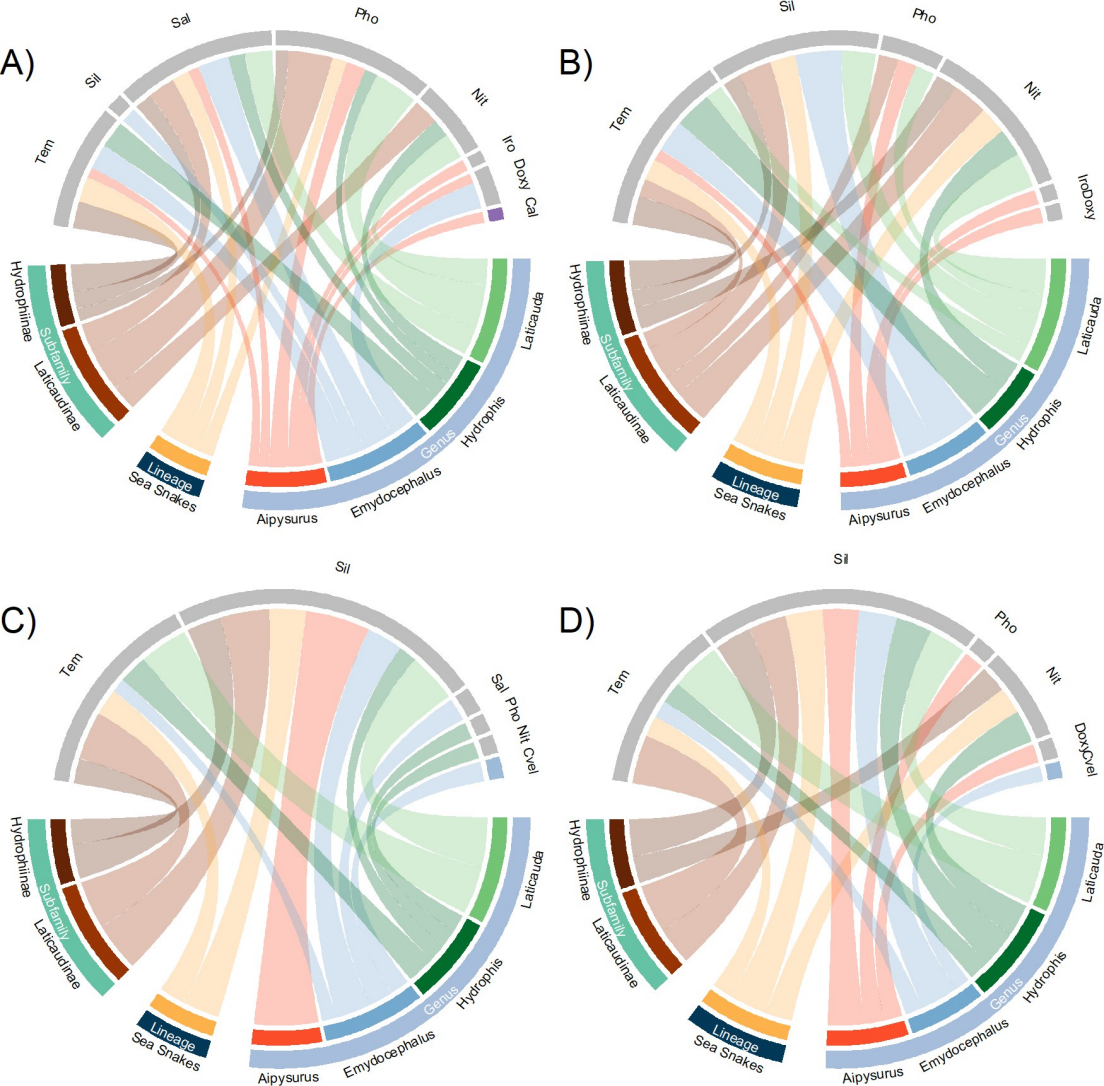
Chord diagram for the relative important variables under the percentage of contribution (PC) and permutation of importance (PI) estimated by MaxEnt and their representation in different taxonomic and spatial resolution levels. A) = PC at 5 arcmin, B) = PI at 5 arcmin, C) = PC at 10 arcmin, D) = PI at 10 arcmin, Nit = nitrates, Pho = phosphates, Sal = salinity, Sil = silicates, Tem = temperature. More connected variables represent that are relatively important variables in more taxonomic and spatial resolution levels comparing to variables less connected.

We present all results of relative importance for each taxonomic level (lineage, subfamily, and genus) in Fig 1 (the results for each species are shown in S1, S2 Figs in S1 File). A summary of the relatively high important variables based on PC and PI as estimated by MaxEnt at 5 and 10 arcmin of spatial resolution is presented in Fig 2.

#### Lineage

The most important variables for sea snakes as a single lineage at a resolution of 5 arcmin, based upon highest PC, were Pho, Sal, and Tem. However, when the importance of variables was permuted (PI), Nit, Sil, and Tem emerged as the most important factors. For both PC and PI at 10 arcmin, Sil and Tem were important variables, while Nit was also important for the PI. Statistically comparing PC against PI, we found differences only in Sal at both resolutions (S3A, S3B Table). When comparing PC between resolutions we found that Sil was statistically different, while comparisons in PI did not show any differences between resolutions (S3C, S3D Table).

#### Subfamily

The patterns of variable importance differed at the subfamily level. For the Hydrophiinae subfamily at 5 arcmin, the most important variables for the PC were Sal and Tem, while the most important for the PI were Nit, Sil and Tem. At 10 arcmin, Sil and Tem were the most important variables for the PC and Nit and Sil for the PI. We note that statistical differences in Sal were recorded between PC and PI at both 5 and 10 arcmin (S3A, S3B Table). For the Laticaudinae subfamily at 5 arcmin, the variables with highest PC were Nit, Pho, and Sal, while the variables with highest PI also included Tem. At 10 arcmin, Sil and Tem were the most important variables for both the PC and PI. Meanwhile, Tem differed between PI at both resolutions (S3D Table).

#### Genus

At the genus level, for *Aipysurus* at 5 arcmin, Cal, Doxy, Iro, Pho, Sal and Tem were the most important variables for the PC, while Doxy, Iro, Pho and Tem were the most important for the PI. At 10 arcmin, Sil was the most important variable for the PC and Doxy, Pho and Sil for the PI. In *Emydocephalus* at 5 arcmin for the PC, Doxy, Sal, Sil and Tem were the most important variables, while Sil and Tem were the most important for the PI. At 10 arcmin, Cvel, Sal, Sil, and Tem were the most important variables for the PC and the same variables omitting Sal for the PI.

For *Hydrophis* at 5 arcmin, Nit, Pho, Sal, and Tem were the most important variables for the PC, while Nit and Tem were the most important for the PI. At 10 arcmin, Nit, Sil, and Tem were the most important variables for both the PC and PI, though Pho also proved important for PC. Additionally, we found differences between Sal when comparing PC against PI at both 5 and 10 arcmin (S3A, S3B Table). For *Laticauda*, the results were similar to those obtained at the subfamily level (containing all species as Laticaudinae; see 3.1.2).

### Variation in the RTN limits

**Fig 3:**
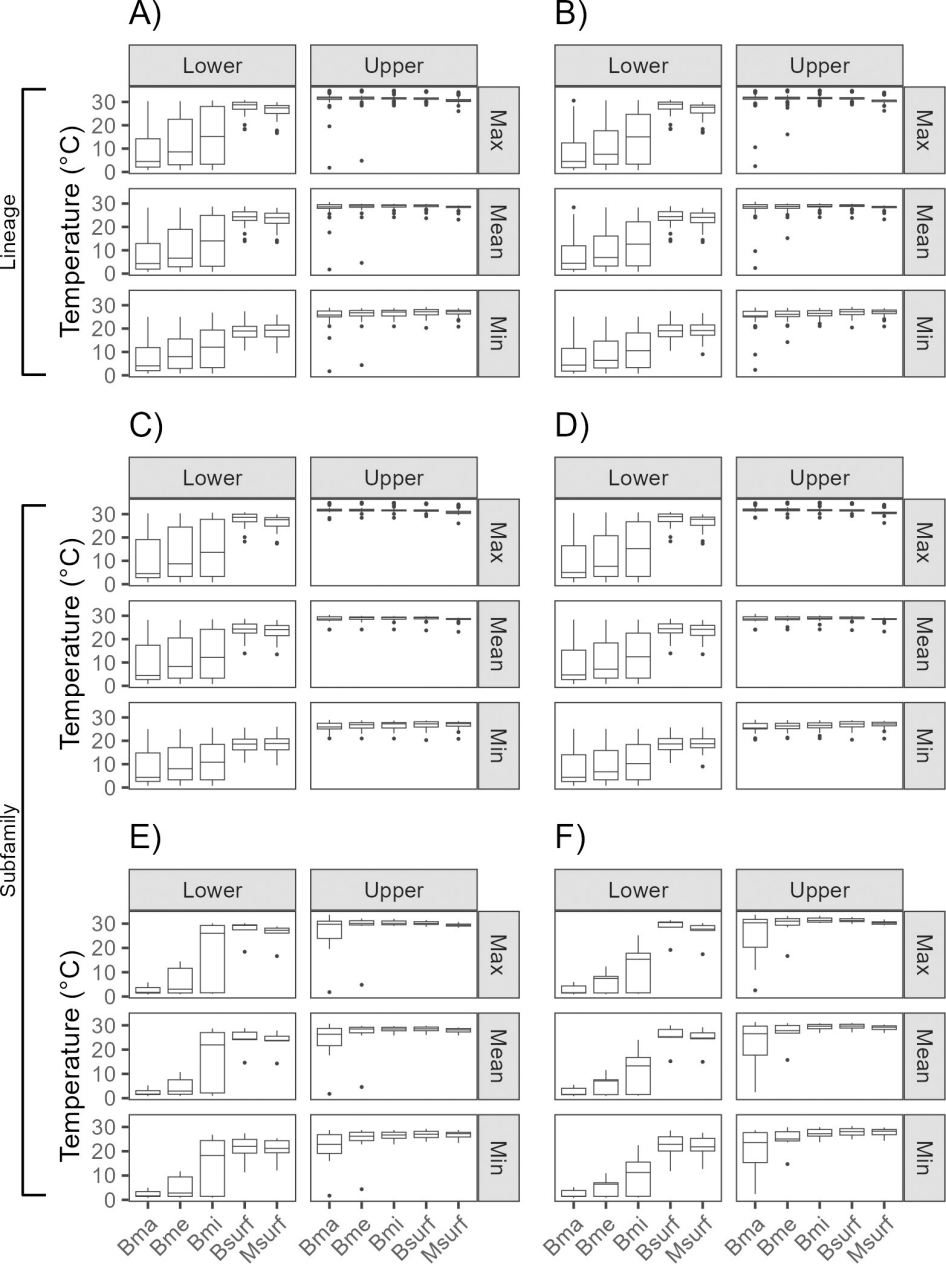
Lower and upper thermal limits of sea snakes at lineage and subfamily taxonomic levels, three temperatures measures, five variable groups and two spatial resolutions. A) = Sea snakes’ lineage at 5 arcmin, B) = Sea snakes’ lineage at 10 arcmin, C) = Hydrophiinae subfamily at 5 arcmin, D) = Hydrophiinae subfamily at 10 arcmin, E) = Laticaudinae subfamily at 5 arcmin, F) = Laticaudinae subfamily at 10 arcmin, Max = maximum temperature, Mean = mean temperature, Min = minimum temperature, Bma = maximum benthic Bio-Oracle, Bme = mean benthic Bio-Oracle, Bmi = minimum benthic Bio-Oracle, Bsurf = superficial Bio-Oracle, Msurf = superficial MARSPEC. *p* values are in S4 Table.

At the lineage level, our results reveal significant differences in the variance of the realized lower thermal limit compared to the realized upper thermal limit across all temperature measurements (mean, maximum, and minimum), regardless of the group of variables used (Bma, Bme, Bmi, Bsurf, and Msurf) or cell resolution (Fig 3A, 3B; S4A, S4B Table). The same pattern was observed in the Hydrophiinae subfamily (Fig 3C, 3D; S4A, S4B Table), whereas for the Laticaudinae subfamily, we found asymmetry with all temperatures in Bmi at both resolutions, only in the minimum temperature measure in Bsurf at both resolutions, and in Bma in minimum temperature at 10 arcmin (Fig 3E, 3F; S4A, S4B Table).

**Fig 4:**
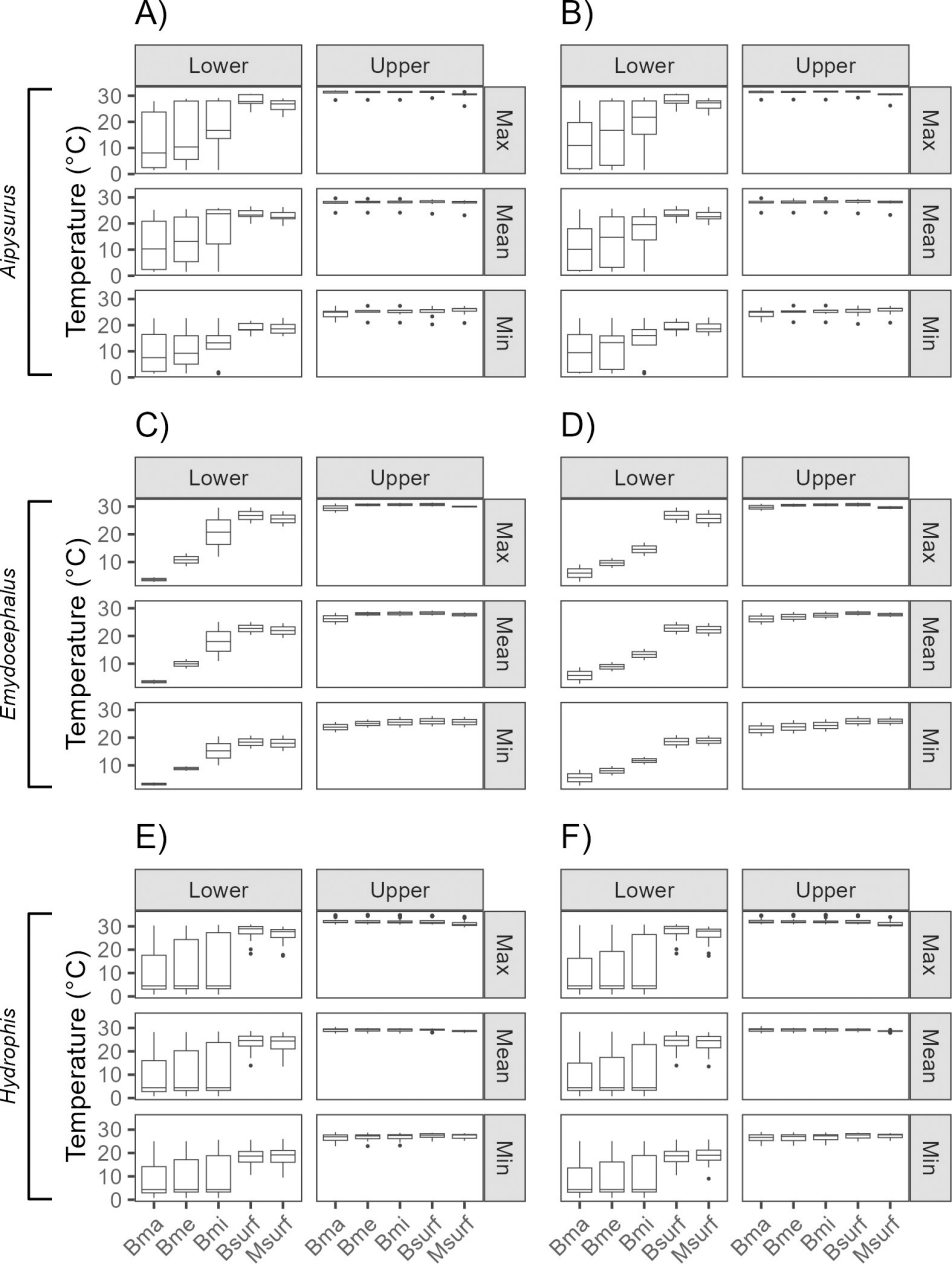
Lower and upper thermal limits of sea snakes at genus taxonomic levels, three temperatures measures, five variable groups and two spatial resolutions. A) *Aipysurus* genus at 5 arcmin, B) = *Aipysurus* genus at 10 arcmin, C) = *Emydocephalus* genus at 5 arcmin, D) = *Emydocephalus* genus at 10 arcmin, E) = *Hydrophis* genus at 5 arcmin, F) = *Hydrophis* genus at 10 arcmin, Max = maximum temperature, Mean = mean temperature, Min = minimum temperature, Bma = maximum benthic Bio-Oracle, Bme = mean benthic Bio-Oracle, Bmi = minimum benthic Bio-Oracle, Bsurf = superficial Bio-Oracle, Msurf = superficial MARSPEC. *p* values are in S4 Table.

Statistically comparing the lower and upper RTN limits at the genus level for *Aipysurus*, we did not observe differences, except in mean and minimum surface temperatures at both resolutions. For *Emydocephalus*, we did not find significant differences between the lower and upper limits at any temperature, repository/depth, or spatial resolution. Conversely, for *Hydrophis*, all temperatures and repository/depths at both spatial resolutions showed statistical differences, which was the same pattern as for the Hydrophiinae subfamily and sea snake lineage as a whole (Fig 4; S4A, S4B Table).

## Discussion

### Overview

Our study adds to the growing body of literature on the ecology of sea snakes and provides further insights into the factors that may influence their distribution patterns. This work utilized correlative models to determine the relationships between sea snakes at multiple taxonomic levels and environmental variables. We examined numerous proximal (direct) predictors, including temperature, salinity, dissolved molecular oxygen, pH, benthic current velocity, and several marine nutrients: calcite, iron, nitrate, phosphate, and silicate, and we found that particular environmental variables correspond with the presence of sea snakes. Previous studies with similar correlative methods for several taxa showed temperature, as well the more distal (indirect) predictors of bathymetry, precipitation and distance to shore to be the critical factors in determining the distribution of marine organisms [3, 20]. We did not include distal variables in our study because they often represent a combination of factors influencing a single variable (e.g., distance to shore is likely associated with temperature, salinity, etc.), which limits their explanatory power for species distribution [see 88]. Instead, we focused on environmental variables that have a more direct relationship with the regulatory and resource factors that define a taxon’s range [see 7]. Of course, certain variables appear to be relevant to almost all taxa, while different faunal groups may have different environmental preferences [3, 6, 20, 89, 90]. When looking specifically at sea snakes, our study confirmed temperature, but also salinity (and certain marine nutrients), to be significant variables in determining their distribution. This is in agreement with the findings on *Hydrophis platurus* [91] in the Tropical Indian and Pacific Oceans, with *H*. *platurus xanthos* in Costa Rica [56], and with sea snakes in Australia [55, 92].

Based on our models, temperature appears to be an important predictor at all taxonomic levels and for most species. Ectothermic organisms, such as reptiles, are particularly sensitive to thermal changes due to their reliance on external sources of heat for regulating body temperature [93–95]. Temperature influences metabolic rates, growth rates, and reproductive success of ectothermic organisms [96], and can ultimately influence species distribution [95, 97]. As such, temperature plays a crucial role in shaping the occurrence patterns of sea snakes. The ranges of sea snakes may even expand during warmer periods and contract during cooler ones [98]. Both elevated and lower temperatures affect sea snake ecology and behavior. Elevated temperatures (33–35°C) reduce survival time and disrupt reproductive cycles; breeding often coincides only with warmer periods [98]. Although sea snakes possess genetic sex determination (GSD) rather than temperature-dependent sex determination (TSD) like many reptiles, the thermal environment may still affect functional sex ratios, as observed in terrestrial snakes [98]. Lower temperatures (~7–16°C) generally decrease feeding efficiency, swimming abilities, and orientation capabilities [98]. Also, those changes generate more frequent surfacing and less efficient breathing patterns [58, 99]. These factors could compromise the ability of true sea snakes to acclimate to long-term increases in water temperature [58, 98, 100], while the wider thermal tolerance sensitivity of amphibious sea kraits could prove beneficial than true sea snakes [57, 98].

Salinity was also an influential variable in our environmental niche modeling. Like temperature, it had an almost-universal effect across lineages. Such results are not surprising, since salinity has been indicated to have a direct effect on the transition to marine life, driving evolution in taxa such as sea snakes [53, 101, 102]. Marine snakes developed specialized salt glands modified from sub-lingual glands to regulate salt efficiently [101, 103, 104], though they maintain a critical dependency on fresh water, including rainfall lenses at the ocean surface [91, 105, 106]. *Laticauda spp*. drink from fresh water sources found on land [105]. Based on their physiological requirements and those of their prey, sea snakes must distribute themselves in areas that facilitate osmotic balance [101], in certain cases avoiding extremely low-saline [100] or high-saline [54] waters. Because in this way salinity can influence their movements [91] it may act as a barrier, while also impacting population health and species diversity [107].

Our sea snake models further indicate that combinations of nutrients such as calcite, iron, nitrate, phosphate, and silicate have relative importance across various taxonomic levels. Nutrient availability is likely to influence the distribution and abundance of sea snake prey [108], as well as the coral reefs that provide habitat for multiple species [98, 109, 110]. This interconnected scenario (availability of nutrients influencing prey types and their habitats) contributes to the diversity of trophic specialization and feeding strategies observed in sea snakes [60, 111, 112], and sometimes even a rapid evolution of head size and body shape [60].

Although our findings provide important insights, caution must be exercised when interpreting the results of correlative models because they cannot establish causality. Some correlations may reflect the direct effects of environmental variables, while others may represent indirect relationships. We used MaxEnt, which can handle collinearity among explanatory variables; however, collinearity may still pose challenges when estimating the importance of explanatory variables [113]. The programmers of MaxEnt incorporated two different metrics (PC and PI) to evaluate the contribution of predictors to the model [84], although there is no consensus in the literature on which is more informative. Most studies use the PC, possibly because it appears first in the algorithm’s results. Halvorsen [114] concluded that the PC is most informative, whereas Searcy and Shaffer [115] suggested that the PI is better for describing the biological properties of species. Further research is necessary to establish causal relationships between environmental factors and the abundance of sea snakes and to gain a definitive understanding of their ecology and behavior.

#### Variance between taxonomic levels

Overall, it appears that variable importance for the presence sea snakes varies depending on which level is evaluated, which species are included, and which methods (including raster resolution) are used. In our model outputs for different lineage and subfamily levels, metrics, and resolutions, temperature consistently showed the greatest influence, followed by salinity and the nutrients silicate, nitrate and phosphate. Another study also found distance to the coast and bathymetry to be relevant variables for predicting the presence of Laticaudinae [108].

When looking at the genus level, our *Aipysurus* models showed dissolved oxygen and iron to be more important than for other groups, though the nutrients calcite, phosphate, and silicate, as well as salinity and temperature, were also influential. Evidence indicates that environmental factors are important in shaping the behavior and distribution of *A*. *laevis* [116]. These factors have been linked to sexual dimorphism [117, 118], potentially related to ecological niche differentiation. Males and females may have adapted morphological differences in head size and body shape through dietary or feeding niche separation; specifically, females exhibit larger body cavities and smaller head sizes, which are associated with a decrease in intra-specific competition [117]. For *Emydocephalus*, we found temperature, silicate, salinity, current velocity and dissolved oxygen to be the most important variables. The relative importance of the different predictors may be influenced by this genus’ trend to occur in discrete patches of suitable habitat (sometimes referred to as “metapopulations”), which are affected by differences in prey abundance, habitat quality, and complexity of reef habitats [119]. Hence, negative trends in population abundance may be correlated to declines in environment resources resulting from anthropogenic modification of the ecosystem [120]. Regarding the importance of temperature, we discovered a discrepancy with a previous study of *E*. *annulatus*. It reported no significant effects of annual thermal variation on population densities, but found that water temperature significantly contributed to the species’ distribution [121]. Further research is necessary to resolve these inconsistencies and to enhance our understanding of the importance of environmental variables, such as salinity and other nutrient variables in the distribution and population dynamics of *Emydocephalus*. The *Hydrophis* genus was found to have almost identical results to the whole lineage, and as indicated above, the genus *Laticauda* is synonymous with the subfamily Laticaudinae; hence, these are not discussed separately.

### RTN limits

According to our research findings, sea snakes demonstrate greater consistency in the variance of their upper thermal limits than in their lower thermal limits. This aligns with the results of previous studies on terrestrial species [122–124] and marine ectotherms [28, 125, 126]. Such results also align with existing evidence that cold tolerance evolves more rapidly than heat tolerance in both endothermic and ectothermic organisms. This phenomenon is attributed to different adaptations related to climatic extremes throughout the evolutionary history of taxa [127]: there is a consistent pattern of heat tolerance, whereas cold tolerance shows significant variability even within species [39, 122, 127]. Sea snakes may too have developed greater plasticity in their lower thermal limits through evolutionary processes [128, 129], resulting in less variation in their upper thermal limits. Moreover, physiology and biophysics may contribute to this asymmetry in tolerances limits and influence RTN limits.

If sea temperatures continue to rise due to climate change, the reduced variability in the upper thermal limits could have significant consequences for sea snakes [122, 130]. Indeed, marine ectotherms have a low physiological acclimatization rate, which makes them particularly susceptible to the effects of climate change [123, 131]. With elongated bodies, sea snakes facilitate rapid heat exchange due to their greater surface in comparison to body mass [132]; however, previous studies have indicated that sea snakes live near their upper thermal limits [98] and are unlikely to evolve increased heat tolerances [122]. Additionally, these thermal niche boundaries are linked to changes in diving and breathing behaviors, leading to more frequent surfacing and less efficient breathing patterns [58, 99]. These factors could compromise the ability of true sea snakes to acclimate to long-term increases in water temperature [58, 98, 100], while greater metabolic thermal tolerance in amphibious sea kraits could prove beneficial to that group [57, 98]. Although our study observed this pattern at the lineage level, we obtained mixed results at the subfamily and genus levels, potentially owing to limited sample size. Other factors, including biotic interactions, geographic barriers, and dispersal processes, may restrict species distribution and affect the capacity to measure complete thermal niches [39, 123, 124].

The consequences of the observed asymmetric realized thermal limits for the physiological performance of sea snakes and their ecological interactions with other species are ecologically significant. Sea snakes are critical predators in marine ecosystems, and alterations in their distribution or abundance can have cascading effects on food webs [133]. Thus, the observed thermal limits of sea snakes have implications not only for their own survival but also for the health and functioning of marine ecosystems. Indeed, our research highlights the importance of conservation efforts and marine management policies that consider the potential vulnerability of sea snakes to changes in sea temperature, including identifying areas that provide thermal refugia for sea snakes, monitoring populations for changes in their distribution or abundance, and implementing strategies that can respond to changing conditions [130], for the benefit of biodiversity. From a research perspective, we note that sample sizes for some subfamilies and genera were relatively small, which may have affected our results, and we encourage future studies to explore the potential for sea snakes to acclimate or adapt to warming temperatures and investigate the mechanisms underlying the observed pattern of asymmetric realized thermal limits. Integrating physiological and genetic data could also help provide a more comprehensive understanding of the thermal ecology of sea snakes and their ability to adapt to climate change.

## Conclusions

Temperature, salinity, and some nutrients (such as nitrates, phosphates, and silicates) appear significant in shaping the distribution of sea snakes. Most sea snakes also demonstrate asymmetry in their realized thermal limits, which is to say, they have accumulated greater plasticity in their lower thermal limits through evolutionary processes with less variation in their upper thermal limits. This pattern is consistent with previous research on ectotherms and other endothermic species [122, 130] and highlights the potentially critical implications for sea snakes under scenarios of sea warming. Marine environmental variables have clear bearing on the lives of sea snakes and ENMs that incorporate them can provide valuable insights for conservation management strategies. They can even help guide searches for previously unknown, potentially endangered, populations [130]. Our findings emphasize the importance of generating scientifically robust ecological niche models at multiple scales, which can guide future marine policies for the protection of these vulnerable and ecologically important reptiles.

## Supporting information

S1 Table Access species-specific data via GBIF using the provided DOI references. This table contains all the DOI facilitated by GBIF to referencing their data.
(PDF)

S2 Table Final model parameterizations are outlined for each species based on spatial resolution. This table contains all the model parametrizations for each species.
(PDF)

S3 Table Non-parametric Wilcoxon test comparing the percentage contribution and permutation importance metrics calculated by MaxEnt at 5–10 arc-minute resolution. This table contains the results of the non-parametric Wilcoxon test of MaxEnt metric across taxa level and spatial resolution.
(PDF)

S4 Table Summary of *p* values for homogeneity of variances of lower and upper realized thermal limits by the minimum, mean, and maximum temperatures across lineage, family, and genus levels at 5 arcmin by non-parametric Fligner-Killeen test. This table contains the results of the homogeneity of variances of thermal limits by the non-parametric Fligner-Killeen test. (PDF).
(PDF)

S1 File Variable contribution estimated by MaxEnt percentage of contribution (PC) and permutation of importance (PI) for each species at 5 and 10 arc-minutes. These figures show the contribution of each species estimated by MaxEnt metrics at both resolutions.
(PDF)
