# Supplementary material for: Global analysis of the influence of environmental variables to explain ecological niches and realized thermal niche boundaries of sea snakes
Source: PLoS One. 2024 Dec 5;19(12):e0310456. doi: 10.1371/journal.pone.0310456 (PMC11620380; doi:10.1371/journal.pone.0310456)
Supplement: S1 Table — This table contains all the DOI facilitated by GBIF to referencing their data. (PDF) [file pone.0310456.s001.pdf]

S1 Table. Access species-specific data via GBIF using the provided DOI references. * = insufficient or incomplete data for modeling purposes. Duplicate species entries on the list signify separate data sets within the GBIF repository pertaining to the same species.

**Spp**	**GBIF_doi**
Aip_apr	10.15468/dl.x4csja
Aip_dub	10.15468/dl.ucfp5i
Aip_eyd	10.15468/dl.pr1sos
Aip_fol	10.15468/dl.cicmdn
Aip_fus	10.15468/dl.j04hyq
Aip_lae	10.15468/dl.kpgna0
Aip_mos	10.15468/dl.jikim1
Aip_poo	10.15468/dl.nr26m5
Aip_ten	10.15468/dl.u2894i
Emy_ann	10.15468/dl.f2od3y
Emy_iji	10.15468/dl.mxls5y
Emy_ora*	10.15468/dl.cvjs69
Hyd_ann	10.15468/dl.sfjksh
Hyd_ano*	10.15468/dl.y7adb9
Hyd_atr	10.15468/dl.dhulhk
Hyd_bel*	10.15468/dl.nkwei4
Hyd_bit*	10.15468/dl.cqk7wf
Hyd_bro	10.15468/dl.365fy0
Hyd_cae	10.15468/dl.xbwxwi
Hyd_can*	10.15468/dl.6vd433
Hyd_cog	10.15468/dl.hhoibz
Hyd_cur	10.15468/dl.bjf4mh
Hyd_cur	10.15468/dl.hrxwav
Hyd_cya	10.15468/dl.fjs04b
Hyd_cze	10.15468/dl.waunc9
Hyd_don*	10.15468/dl.xq4we8

Hyd_ele	10.15468/dl.ycskxi
Hyd_fas	10.15468/dl.1wnf1w
Hyd_fas	10.15468/dl.yjeccr
Hyd_gra	10.15468/dl.tptjxh
Hyd_gra	0.15468/dl.f9tww4
Hyd_har	10.15468/dl.t3wvgo
Hyd_hen*	10.15468/dl.ufdeh8
Hyd_ino	10.15468/dl.kzrprh
Hyd_jer	10.15468/dl.u8stiw
Hyd_kin	10.15468/dl.zwcdoj
Hyd_klo	10.15468/dl.hali50
Hyd_lab*	10.15468/dl.vps3vg
Hyd_lam*	10.15468/dl.d4qr5t
Hyd_lap	10.15468/dl.yumdho
Hyd_mac	10.15468/dl.lyylzg
Hyd_maj	10.15468/dl.hzuwyy
Hyd_maj	10.15468/dl.hrf2jj
Hyd_mam*	10.15468/dl.53cz5j
Hyd_melanoc	10.15468/dl.8jae34
Hyd_melanos	10.15468/dl.jxfcd5
Hyd_nig	10.15468/dl.xy7cem
Hyd_obs	10.15468/dl.9mzxev
Hyd_oce	10.15468/dl.0r8oar
Hyd_orn	10.15468/dl.0unzdx
Hyd_pach*	10.15468/dl.tdvqdz
Hyd_pac	10.15468/dl.1k4wyr
Hyd_par*	10.15468/dl.st94qu
Hyd_per	10.15468/dl.8iwxch
Hyd_per	10.15468/dl.cdbs7o
Hyd_pla	10.15468/dl.sxyyau
Hyd_sch	10.15468/dl.uhhuf2

Hyd_sem*	10.15468/dl.ggcvxz
Hyd_sib*	10.15468/dl.v7697p
Hyd_spi	10.15468/dl.csaxez
Hyd_sto	10.15468/dl.cza1cr
Hyd_str*	10.15468/dl.bsjb57
Hyd_tor	10.15468/dl.3qikbk
Hyd_vip	10.15468/dl.tmeusc
Hyd_vip	10.15468/dl.ttwxlk
Hyd_vor*	10.15468/dl.5ryxyc
Hyd_zwe	10.15468/dl.7sydu1
Lat_col	10.15468/dl.hnvfjv
Lat_cro	10.15468/dl.kvtxn0
Lat_fro	10.15468/dl.gg5juy
Lat_gui*	10.15468/dl.dqppbw
Lat_lat	10.15468/dl.hr6lov
Lat_sai	10.15468/dl.uk80jf
Lat_sch	10.15468/dl.zfxk5a
Lat_sem	10.15468/dl.nn22aa
