# Supplementary material for: Global analysis of the influence of environmental variables to explain ecological niches and realized thermal niche boundaries of sea snakes
Source: PLoS One. 2024 Dec 5;19(12):e0310456. doi: 10.1371/journal.pone.0310456 (PMC11620380; doi:10.1371/journal.pone.0310456)
Supplement: S2 Table — This table contains all the model parametrizations for each species. (PDF) [file pone.0310456.s002.pdf]

S2 Table. Final model parameterizations are outlined for each species based on spatial resolution. RF = regularization multiplier, FC = feature classes, _5 = 5 arcmin, _10 = 10 arcmin, l = linear, p = product, q = quadratic, Bma = Bio-Oracle benthic maximum depth, Bme = Bio-Oracle benthic mean depth, Bmi = Bio-Oracle benthic minimum depth, Bsurf = Bio-Oracle surface, Msurf = MARSPEC surface. - denotes that not equal models were performed between spatial resolutions.

Family	Genus	Species	RM_5	FC_5	Set_5	RM_10	FC_10	Set_10
Hydrophiinae	*Aipysurus*	*Aip_apr*	0.10	l	Bma	0.10	qp	Bme
Hydrophiinae	*Aipysurus*	*Aip_apr*	0.25	l	Bma	0.10	lqp	Bme
Hydrophiinae	*Aipysurus*	*Aip_apr*	0.50	l	Bma	0.10	lp	Bma
Hydrophiinae	*Aipysurus*	*Aip_apr*	0.50	lp	Bma	0.25	l	Bma
Hydrophiinae	*Aipysurus*	*Aip_apr*	0.50	lqp	Bma	-	-	-
Hydrophiinae	*Aipysurus*	*Aip_apr*	0.75	lp	Bma	-	-	-
Hydrophiinae	*Aipysurus*	*Aip_apr*	0.75	lqp	Bma	-	-	-
Hydrophiinae	*Aipysurus*	*Aip_dub*	0.25	p	Bmi	1	lqp	Bmi
Hydrophiinae	*Aipysurus*	*Aip_dub*	0.25	q	Bmi	-	-	-
Hydrophiinae	*Aipysurus*	*Aip_dub*	0.5	q	Bmi	-	-	-
Hydrophiinae	*Aipysurus*	*Aip_dub*	0.75	q	Bmi	-	-	-
Hydrophiinae	*Aipysurus*	*Aip_dub*	1	q	Bmi	-	-	-
Hydrophiinae	*Aipysurus*	*Aip_eyd*	2	lq	Bsurf	1	qp	Bsurf
Hydrophiinae	*Aipysurus*	*Aip_fol*	0.10	lp	Msurf	0.10	lqp	Bme
Hydrophiinae	*Aipysurus*	*Aip_fol*	0.10	lqp	Msurf	-	-	-

Hydrophiinae	*Aipysurus*	*Aip_fol*	0.10	p	Msurf	-	-	-
Hydrophiinae	*Aipysurus*	*Aip_fol*	0.10	qp	Msurf	-	-	-
Hydrophiinae	*Aipysurus*	*Aip_fol*	0.25	l	Msurf	-	-	-
Hydrophiinae	*Aipysurus*	*Aip_fol*	0.25	lq	Bme	-	-	-
Hydrophiinae	*Aipysurus*	*Aip_fol*	0.25	q	Msurf	-	-	-
Hydrophiinae	*Aipysurus*	*Aip_fol*	0.25	qp	Msurf	-	-	-
Hydrophiinae	*Aipysurus*	*Aip_fol*	0.50	l	Msurf	-	-	-
Hydrophiinae	*Aipysurus*	*Aip_fol*	0.50	lq	Msurf	-	-	-
Hydrophiinae	*Aipysurus*	*Aip_fol*	0.75	lp	Bme	-	-	-
Hydrophiinae	*Aipysurus*	*Aip_fol*	0.75	p	Bme	-	-	-
Hydrophiinae	*Aipysurus*	*Aip_fus*	1	lq	Bmi	0.10	l	Bmi
Hydrophiinae	*Aipysurus*	*Aip_fus*	2	lq	Bmi	0.25	l	Bmi
Hydrophiinae	*Aipysurus*	*Aip_fus*	-	-	-	0.50	q	Bmi
Hydrophiinae	*Aipysurus*	*Aip_fus*	-	-	-	0.75	q	Bmi
Hydrophiinae	*Aipysurus*	*Aip_lae*	0.10	lp	Bmi	0.50	q	Bsurf
Hydrophiinae	*Aipysurus*	*Aip_lae*	-	-	-	0.75	q	Bsurf
Hydrophiinae	*Aipysurus*	*Aip_mos*	0.50	p	Bsurf	2	l	Bsurf
Hydrophiinae	*Aipysurus*	*Aip_mos*	-	-	-	3	l	Bsurf
Hydrophiinae	*Aipysurus*	*Aip_poo*	0.10	lq	Bmi	4	lp	Bma
Hydrophiinae	*Aipysurus*	*Aip_poo*	0.75	l	Msurf	4	lqp	Bma
Hydrophiinae	*Aipysurus*	*Aip_poo*	0.75	lp	Msurf	-	-	-

Hydrophiinae	*Aipysurus*	*Aip_poo*	0.75	q	Msurf	-	-	-
Hydrophiinae	*Aipysurus*	*Aip_poo*	0.75	qp	Msurf	-	-	-
Hydrophiinae	*Aipysurus*	*Aip_poo*	1	l	Msurf	-	-	-
Hydrophiinae	*Aipysurus*	*Aip_poo*	1	q	Msurf	-	-	-
Hydrophiinae	*Aipysurus*	*Aip_poo*	4	lp	Bma	-	-	-
Hydrophiinae	*Aipysurus*	*Aip_poo*	4	lqp	Bma	-	-	-
Hydrophiinae	*Aipysurus*	*Aip_poo*	4	qp	Bma	-	-	-
Hydrophiinae	*Aipysurus*	*Aip_ten*	0.75	qp	Bme	2	q	Bme
Hydrophiinae	*Aipysurus*	*Aip_ten*	2	q	Bma	2	qp	Bme
Hydrophiinae	*Aipysurus*	*Aip_ten*	2	qp	Bma	2	lqp	Bme
Hydrophiinae	*Aipysurus*	*Aip_ten*	2	lqp	Bma	2	p	Bme
Hydrophiinae	*Aipysurus*	*Aip_ten*	2	qp	Bme	2	lp	Bme
Hydrophiinae	*Aipysurus*	*Aip_ten*	2	lqp	Bme	2	q	Bmi
Hydrophiinae	*Aipysurus*	*Aip_ten*	2	p	Bme	2	qp	Bmi
Hydrophiinae	*Aipysurus*	*Aip_ten*	2	lp	Bme	2	lqp	Bmi
Hydrophiinae	*Aipysurus*	*Aip_ten*	3	l	Bma	3	l	Bme
Hydrophiinae	*Aipysurus*	*Aip_ten*	3	lq	Bma	3	lq	Bme
Hydrophiinae	*Aipysurus*	*Aip_ten*	3	q	Bma	3	l	Bmi
Hydrophiinae	*Aipysurus*	*Aip_ten*	3	qp	Bma	3	lq	Bmi
Hydrophiinae	*Aipysurus*	*Aip_ten*	3	lqp	Bma	3	q	Bme
Hydrophiinae	*Aipysurus*	*Aip_ten*	3	l	Bme	3	qp	Bme

Hydrophiinae	*Aipysurus*	*Aip_ten*	3	lq	Bme	3	lqp	Bme
Hydrophiinae	*Aipysurus*	*Aip_ten*	3	p	Bma	3	lp	Bme
Hydrophiinae	*Aipysurus*	*Aip_ten*	3	lp	Bma	3	p	Bme
Hydrophiinae	*Aipysurus*	*Aip_ten*	4	l	Bma	3	qp	Bmi
Hydrophiinae	*Aipysurus*	*Aip_ten*	4	lq	Bma	3	lqp	Bmi
Hydrophiinae	*Aipysurus*	*Aip_ten*	4	q	Bma	3	q	Bmi
Hydrophiinae	*Aipysurus*	*Aip_ten*	4	qp	Bma	3	p	Bmi
Hydrophiinae	*Aipysurus*	*Aip_ten*	4	lqp	Bma	3	lp	Bmi
Hydrophiinae	*Aipysurus*	*Aip_ten*	4	l	Bme	4	l	Bme
Hydrophiinae	*Aipysurus*	*Aip_ten*	4	lq	Bme	4	lq	Bme
Hydrophiinae	*Aipysurus*	*Aip_ten*	-	-	-	4	l	Bmi
Hydrophiinae	*Aipysurus*	*Aip_ten*	-	-	-	4	lq	Bmi
Hydrophiinae	*Aipysurus*	*Aip_ten*	-	-	-	4	q	Bme
Hydrophiinae	*Aipysurus*	*Aip_ten*	-	-	-	4	qp	Bme
Hydrophiinae	*Aipysurus*	*Aip_ten*	-	-	-	4	lqp	Bme
Hydrophiinae	*Aipysurus*	*Aip_ten*	-	-	-	4	p	Bme
Hydrophiinae	*Aipysurus*	*Aip_ten*	-	-	-	4	lp	Bme
Hydrophiinae	*Aipysurus*	*Aip_ten*	-	-	-	4	p	Bmi
Hydrophiinae	*Aipysurus*	*Aip_ten*	-	-	-	4	lp	Bmi
Hydrophiinae	*Emydocephalus*	*Emy_ann*	0.5	p	Bmi	0.25	qp	Bma
Hydrophiinae	*Emydocephalus*	*Emy_ann*	-	-	-	0.25	lq	Bma

Hydrophiinae	*Emydocephalus*	*Emy_ann*	-	-	-	0.75	qp	Bma
Hydrophiinae	*Emydocephalus*	*Emy_iji*	0.25	l	Bme	0.10	q	Bsurf
Hydrophiinae	*Emydocephalus*	*Emy_iji*	0.25	p	Bme	0.25	q	Bsurf
Hydrophiinae	*Emydocephalus*	*Emy_iji*	0.25	lp	Bme	0.50	q	Bsurf
Hydrophiinae	*Emydocephalus*	*Emy_iji*	0.50	l	Bme	-	-	-
Hydrophiinae	*Emydocephalus*	*Emy_iji*	0.50	p	Bme	-	-	-
Hydrophiinae	*Emydocephalus*	*Emy_iji*	0.50	lp	Bme	-	-	-
Hydrophiinae	*Emydocephalus*	*Emy_iji*	0.50	qp	Bme	-	-	-
Hydrophiinae	*Emydocephalus*	*Emy_iji*	0.50	lqp	Bme	-	-	-
Hydrophiinae	*Emydocephalus*	*Emy_iji*	0.75	l	Bme	-	-	-
Hydrophiinae	*Emydocephalus*	*Emy_iji*	0.75	p	Bme	-	-	-
Hydrophiinae	*Emydocephalus*	*Emy_iji*	0.75	qp	Bme	-	-	-
Hydrophiinae	*Emydocephalus*	*Emy_iji*	1	l	Bme	-	-	-
Hydrophiinae	*Emydocephalus*	*Emy_iji*	1	p	Bme	-	-	-
Hydrophiinae	*Emydocephalus*	*Emy_iji*	1	qp	Bme	-	-	-
Hydrophiinae	*Emydocephalus*	*Emy_iji*	2	lq	Bme	-	-	-
Hydrophiinae	*Emydocephalus*	*Emy_iji*	2	l	Bme	-	-	-
Hydrophiinae	*Hydrophis*	*Hyd_atr*	1	lq	Bma	0.25	p	Bmi
Hydrophiinae	*Hydrophis*	*Hyd_atr*	2	q	Bma	0.75	p	Bme
Hydrophiinae	*Hydrophis*	*Hyd_atr*	2	lqp	Bma	1	lqp	Bma
Hydrophiinae	*Hydrophis*	*Hyd_atr*	2	qp	Bma	1	p	Bma

Hydrophiinae	*Hydrophis*	*Hyd_atr*	3	lq	Bma	1	p	Bme
Hydrophiinae	*Hydrophis*	*Hyd_atr*	3	q	Bma	1	p	Bmi
Hydrophiinae	*Hydrophis*	*Hyd_atr*	4	lq	Bma	2	lq	Bma
Hydrophiinae	*Hydrophis*	*Hyd_atr*	4	q	Bma	2	q	Bma
Hydrophiinae	*Hydrophis*	*Hyd_atr*	-	-	-	2	lqp	Bma
Hydrophiinae	*Hydrophis*	*Hyd_atr*	-	-	-	2	qp	Bma
Hydrophiinae	*Hydrophis*	*Hyd_atr*	-	-	-	2	l	Bma
Hydrophiinae	*Hydrophis*	*Hyd_atr*	-	-	-	3	lq	Bma
Hydrophiinae	*Hydrophis*	*Hyd_atr*	-	-	-	3	q	Bma
Hydrophiinae	*Hydrophis*	*Hyd_atr*	-	-	-	3	lqp	Bma
Hydrophiinae	*Hydrophis*	*Hyd_atr*	-	-	-	3	qp	Bma
Hydrophiinae	*Hydrophis*	*Hyd_atr*	-	-	-	4	lq	Bma
Hydrophiinae	*Hydrophis*	*Hyd_atr*	-	-	-	4	q	Bma
Hydrophiinae	*Hydrophis*	*Hyd_bel*	0.25	lqp	Bmi	0.25	lq	Bma
Hydrophiinae	*Hydrophis*	*Hyd_bel*	0.25	qp	Bmi	-	-	-
Hydrophiinae	*Hydrophis*	*Hyd_bel*	0.50	lp	Bmi	-	-	-
Hydrophiinae	*Hydrophis*	*Hyd_bel*	0.50	lq	Bmi	-	-	-
Hydrophiinae	*Hydrophis*	*Hyd_bel*	0.50	q	Bmi	-	-	-
Hydrophiinae	*Hydrophis*	*Hyd_bel*	0.50	lqp	Bmi	-	-	-
Hydrophiinae	*Hydrophis*	*Hyd_bel*	0.75	l	Bmi	-	-	-
Hydrophiinae	*Hydrophis*	*Hyd_bel*	0.75	lp	Bmi	-	-	-

Hydrophiinae	*Hydrophis*	*Hyd_bel*	0.75	lqp	Bmi	-	-	-
Hydrophiinae	*Hydrophis*	*Hyd_bel*	0.75	qp	Bmi	-	-	-
Hydrophiinae	*Hydrophis*	*Hyd_bel*	0.75	lq	Bmi	-	-	-
Hydrophiinae	*Hydrophis*	*Hyd_bel*	0.75	q	Bmi	-	-	-
Hydrophiinae	*Hydrophis*	*Hyd_bel*	1	l	Bmi	-	-	-
Hydrophiinae	*Hydrophis*	*Hyd_bel*	1	lp	Bmi	-	-	-
Hydrophiinae	*Hydrophis*	*Hyd_bel*	1	lqp	Bmi	-	-	-
Hydrophiinae	*Hydrophis*	*Hyd_bel*	1	qp	Bmi	-	-	-
Hydrophiinae	*Hydrophis*	*Hyd_bel*	1	lq	Bmi	-	-	-
Hydrophiinae	*Hydrophis*	*Hyd_bel*	2	lq	Bmi	-	-	-
Hydrophiinae	*Hydrophis*	*Hyd_bel*	2	l	Bmi	-	-	-
Hydrophiinae	*Hydrophis*	*Hyd_bel*	2	lp	Bmi	-	-	-
Hydrophiinae	*Hydrophis*	*Hyd_bel*	2	lqp	Bmi	-	-	-
Hydrophiinae	*Hydrophis*	*Hyd_bel*	2	q	Bmi	-	-	-
Hydrophiinae	*Hydrophis*	*Hyd_bel*	3	lq	Bmi	-	-	-
Hydrophiinae	*Hydrophis*	*Hyd_bel*	3	l	Bmi	-	-	-
Hydrophiinae	*Hydrophis*	*Hyd_bel*	3	q	Bmi	-	-	-
Hydrophiinae	*Hydrophis*	*Hyd_bel*	3	lp	Bmi	-	-	-
Hydrophiinae	*Hydrophis*	*Hyd_bel*	3	lqp	Bmi	-	-	-
Hydrophiinae	*Hydrophis*	*Hyd_bel*	4	lq	Bmi	-	-	-
Hydrophiinae	*Hydrophis*	*Hyd_bel*	4	l	Bmi	-	-	-

Hydrophiinae	*Hydrophis*	*Hyd_bro*	2	q	Bmi	3	p	Bme
Hydrophiinae	*Hydrophis*	*Hyd_bro*	-	-	-	3	lp	Bme
Hydrophiinae	*Hydrophis*	*Hyd_bro*	-	-	-	4	p	Bme
Hydrophiinae	*Hydrophis*	*Hyd_bro*	-	-	-	4	lp	Bme
Hydrophiinae	*Hydrophis*	*Hyd_cae*	0.50	lq	Bmi	0.10	lq	Bmi
Hydrophiinae	*Hydrophis*	*Hyd_cae*	0.75	lq	Bmi	0.50	lq	Bmi
Hydrophiinae	*Hydrophis*	*Hyd_cae*	1	lq	Bmi	0.75	l	Bmi
Hydrophiinae	*Hydrophis*	*Hyd_cae*	-	-	-	0.75	lq	Bmi
Hydrophiinae	*Hydrophis*	*Hyd_cae*	-	-	-	1	lq	Bmi
Hydrophiinae	*Hydrophis*	*Hyd_cae*	-	-	-	3	lq	Bmi
Hydrophiinae	*Hydrophis*	*Hyd_cog*	2	lp	Bma	2	p	Bsurf
Hydrophiinae	*Hydrophis*	*Hyd_cog*	2	lp	Bme	2	lp	Bsurf
Hydrophiinae	*Hydrophis*	*Hyd_cog*	2	lqp	Bma	2	qp	Bsurf
Hydrophiinae	*Hydrophis*	*Hyd_cog*	2	lqp	Bme	2	lqp	Bsurf
Hydrophiinae	*Hydrophis*	*Hyd_cog*	2	qp	Bma	3	lq	Bsurf
Hydrophiinae	*Hydrophis*	*Hyd_cog*	2	qp	Bme	-	-	-
Hydrophiinae	*Hydrophis*	*Hyd_cog*	4	lq	Bma	-	-	-
Hydrophiinae	*Hydrophis*	*Hyd_cog*	4	lq	Bme	-	-	-
Hydrophiinae	*Hydrophis*	*Hyd_cur*	2	p	Bmi	0.10	lp	Msurf
Hydrophiinae	*Hydrophis*	*Hyd_cya*	0.75	p	Bmi	0.50	lp	Msurf
Hydrophiinae	*Hydrophis*	*Hyd_cya*	0.75	qp	Bmi	0.75	lq	Msurf

Hydrophiinae	*Hydrophis*	*Hyd_cya*	0.75	lp	Bmi	0.75	qp	Msurf
Hydrophiinae	*Hydrophis*	*Hyd_cya*	0.75	lqp	Bmi	0.75	lp	Msurf
Hydrophiinae	*Hydrophis*	*Hyd_cya*	1	p	Bmi	1	q	Msurf
Hydrophiinae	*Hydrophis*	*Hyd_cya*	1	lp	Bmi	2	q	Msurf
Hydrophiinae	*Hydrophis*	*Hyd_cya*	1	lqp	Bmi	2	lq	Msurf
Hydrophiinae	*Hydrophis*	*Hyd_cya*	1	qp	Bmi	-	-	-
Hydrophiinae	*Hydrophis*	*Hyd_cze*	0.10	p	Msurf	0.10	l	Msurf
Hydrophiinae	*Hydrophis*	*Hyd_cze*	0.25	p	Msurf	0.10	lp	Msurf
Hydrophiinae	*Hydrophis*	*Hyd_cze*	0.50	p	Msurf	0.10	qp	Msurf
Hydrophiinae	*Hydrophis*	*Hyd_cze*	0.75	p	Msurf	0.10	q	Msurf
Hydrophiinae	*Hydrophis*	*Hyd_cze*	2	l	Msurf	0.25	l	Msurf
Hydrophiinae	*Hydrophis*	*Hyd_cze*	3	q	Msurf	0.25	q	Msurf
Hydrophiinae	*Hydrophis*	*Hyd_cze*	-	-	-	0.25	lp	Msurf
Hydrophiinae	*Hydrophis*	*Hyd_cze*	-	-	-	0.25	qp	Msurf
Hydrophiinae	*Hydrophis*	*Hyd_cze*	-	-	-	0.25	lqp	Msurf
Hydrophiinae	*Hydrophis*	*Hyd_cze*	-	-	-	0.50	lq	Msurf
Hydrophiinae	*Hydrophis*	*Hyd_cze*	-	-	-	0.50	l	Msurf
Hydrophiinae	*Hydrophis*	*Hyd_cze*	-	-	-	0.50	q	Msurf
Hydrophiinae	*Hydrophis*	*Hyd_cze*	-	-	-	0.50	qp	Msurf
Hydrophiinae	*Hydrophis*	*Hyd_cze*	-	-	-	0.50	lqp	Msurf
Hydrophiinae	*Hydrophis*	*Hyd_cze*	-	-	-	0.50	lp	Msurf

Hydrophiinae	*Hydrophis*	*Hyd_cze*	-	-	-	0.75	lq	Msurf
Hydrophiinae	*Hydrophis*	*Hyd_cze*	-	-	-	0.75	q	Msurf
Hydrophiinae	*Hydrophis*	*Hyd_cze*	-	-	-	0.75	l	Msurf
Hydrophiinae	*Hydrophis*	*Hyd_cze*	-	-	-	0.75	qp	Msurf
Hydrophiinae	*Hydrophis*	*Hyd_cze*	-	-	-	0.75	lp	Msurf
Hydrophiinae	*Hydrophis*	*Hyd_cze*	-	-	-	1	lq	Msurf
Hydrophiinae	*Hydrophis*	*Hyd_cze*	-	-	-	1	q	Msurf
Hydrophiinae	*Hydrophis*	*Hyd_cze*	-	-	-	1	l	Msurf
Hydrophiinae	*Hydrophis*	*Hyd_ele*	0.10	qp	Bma	0.25	qp	Bma
Hydrophiinae	*Hydrophis*	*Hyd_fas*	0.75	lp	Bmi	0.75	lq	Bmi
Hydrophiinae	*Hydrophis*	*Hyd_fas*	1	p	Bmi	0.75	p	Bmi
Hydrophiinae	*Hydrophis*	*Hyd_fas*	1	qp	Bmi	1	qp	Bmi
Hydrophiinae	*Hydrophis*	*Hyd_fas*	1	lqp	Bmi	1	lqp	Bmi
Hydrophiinae	*Hydrophis*	*Hyd_fas*	1	q	Bma	2	lq	Bmi
Hydrophiinae	*Hydrophis*	*Hyd_fas*	1	p	Bme	3	lq	Bmi
Hydrophiinae	*Hydrophis*	*Hyd_fas*	2	p	Bme	-	-	-
Hydrophiinae	*Hydrophis*	*Hyd_fas*	2	lp	Bmi	-	-	-
Hydrophiinae	*Hydrophis*	*Hyd_fas*	3	lp	Bmi	-	-	-
Hydrophiinae	*Hydrophis*	*Hyd_fas*	4	lq	Bmi	-	-	-
Hydrophiinae	*Hydrophis*	*Hyd_gra*	2	p	Bma	0.10	l	Bme
Hydrophiinae	*Hydrophis*	*Hyd_gra*	2	lp	Bma	0.50	l	Bmi

Hydrophiinae	*Hydrophis*	*Hyd_gra*	3	lp	Bma	0.75	p	Bme
Hydrophiinae	*Hydrophis*	*Hyd_gra*	3	qp	Bma	0.75	lp	Bme
Hydrophiinae	*Hydrophis*	*Hyd_gra*	3	lqp	Bma	1	p	Bme
Hydrophiinae	*Hydrophis*	*Hyd_gra*	3	p	Bma	1	lp	Bme
Hydrophiinae	*Hydrophis*	*Hyd_gra*	4	p	Bma	-	-	-
Hydrophiinae	*Hydrophis*	*Hyd_gra*	4	lp	Bma	-	-	-
Hydrophiinae	*Hydrophis*	*Hyd_gra*	4	qp	Bma	-	-	-
Hydrophiinae	*Hydrophis*	*Hyd_gra*	4	lqp	Bma	-	-	-
Hydrophiinae	*Hydrophis*	*Hyd_har*	0.25	qp	Msurf	0.10	q	Msurf
Hydrophiinae	*Hydrophis*	*Hyd_har*	-	-	-	0.25	q	Msurf
Hydrophiinae	*Hydrophis*	*Hyd_ino*	0.50	lqp	Bma	0.50	qp	Bma
Hydrophiinae	*Hydrophis*	*Hyd_ino*	0.50	qp	Bma	0.50	lqp	Bma
Hydrophiinae	*Hydrophis*	*Hyd_ino*	0.50	lp	Bma	0.50	qp	Bme
Hydrophiinae	*Hydrophis*	*Hyd_ino*	0.50	qp	Bme	0.50	lqp	Bme
Hydrophiinae	*Hydrophis*	*Hyd_ino*	0.50	lqp	Bme	0.50	p	Bma
Hydrophiinae	*Hydrophis*	*Hyd_ino*	0.50	p	Bma	0.50	lp	Bma
Hydrophiinae	*Hydrophis*	*Hyd_ino*	0.75	qp	Bma	0.50	qp	Bmi
Hydrophiinae	*Hydrophis*	*Hyd_ino*	0.75	lqp	Bma	0.50	lqp	Bmi
Hydrophiinae	*Hydrophis*	*Hyd_ino*	0.75	lqp	Bme	0.50	p	Bme
Hydrophiinae	*Hydrophis*	*Hyd_ino*	0.75	qp	Bme	0.50	lp	Bme
Hydrophiinae	*Hydrophis*	*Hyd_ino*	1	lq	Bma	0.75	qp	Bma

Hydrophiinae	*Hydrophis*	*Hyd_ino*	1	qp	Bma	0.75	lqp	Bma
Hydrophiinae	*Hydrophis*	*Hyd_ino*	1	lqp	Bma	0.75	qp	Bme
Hydrophiinae	*Hydrophis*	*Hyd_ino*	1	lq	Bme	0.75	lqp	Bme
Hydrophiinae	*Hydrophis*	*Hyd_ino*	1	l	Bma	0.75	lp	Bma
Hydrophiinae	*Hydrophis*	*Hyd_ino*	2	q	Bsurf	0.75	p	Bma
Hydrophiinae	*Hydrophis*	*Hyd_ino*	2	p	Bma	1	lq	Bma
Hydrophiinae	*Hydrophis*	*Hyd_ino*	2	lp	Bma	1	qp	Bme
Hydrophiinae	*Hydrophis*	*Hyd_ino*	3	l	Bma	1	l	Bma
Hydrophiinae	*Hydrophis*	*Hyd_ino*	3	l	Bme	1	qp	Bma
Hydrophiinae	*Hydrophis*	*Hyd_ino*	4	l	Bma	1	lqp	Bma
Hydrophiinae	*Hydrophis*	*Hyd_ino*	-	-	-	1	q	Bma
Hydrophiinae	*Hydrophis*	*Hyd_ino*	-	-	-	2	q	Bsurf
Hydrophiinae	*Hydrophis*	*Hyd_ino*	-	-	-	2	p	Bma
Hydrophiinae	*Hydrophis*	*Hyd_ino*	-	-	-	2	lp	Bma
Hydrophiinae	*Hydrophis*	*Hyd_ino*	-	-	-	3	l	Bma
Hydrophiinae	*Hydrophis*	*Hyd_ino*	-	-	-	3	q	Bsurf
Hydrophiinae	*Hydrophis*	*Hyd_ino*	-	-	-	4	l	Bma
Hydrophiinae	*Hydrophis*	*Hyd_jer*	4	lqp	Bsurf	0.10	p	Bsurf
Hydrophiinae	*Hydrophis*	*Hyd_jer*	4	qp	Bsurf	-	-	-
Hydrophiinae	*Hydrophis*	*Hyd_jer*	4	l	Bsurf	-	-	-
Hydrophiinae	*Hydrophis*	*Hyd_kin*	0.50	lqp	Msurf	4	p	Msurf

Hydrophiinae	*Hydrophis*	*Hyd_kin*	0.50	qp	Msurf	-	-	-
Hydrophiinae	*Hydrophis*	*Hyd_kin*	0.75	lqp	Msurf	-	-	-
Hydrophiinae	*Hydrophis*	*Hyd_kin*	1	lqp	Msurf	-	-	-
Hydrophiinae	*Hydrophis*	*Hyd_klo*	0.10	p	Bmi	0.10	p	Bmi
Hydrophiinae	*Hydrophis*	*Hyd_klo*	0.25	p	Bmi	0.25	p	Bsurf
Hydrophiinae	*Hydrophis*	*Hyd_klo*	-	-	-	0.25	p	Bmi
Hydrophiinae	*Hydrophis*	*Hyd_klo*	-	-	-	0.50	p	Bmi
Hydrophiinae	*Hydrophis*	*Hyd_klo*	-	-	-	0.50	p	Bsurf
Hydrophiinae	*Hydrophis*	*Hyd_klo*	-	-	-	0.50	lp	Bsurf
Hydrophiinae	*Hydrophis*	*Hyd_lap*	3	p	Bsurf	2	p	Bsurf
Hydrophiinae	*Hydrophis*	*Hyd_mac*	3	lqp	Bmi	2	lqp	Bmi
Hydrophiinae	*Hydrophis*	*Hyd_mac*	3	qp	Bmi	2	qp	Bmi
Hydrophiinae	*Hydrophis*	*Hyd_maj*	0.25	lqp	Bmi	0.75	p	Bmi
Hydrophiinae	*Hydrophis*	*Hyd_melanoc*	0.10	lq	Bsurf	0.10	lq	Bme
Hydrophiinae	*Hydrophis*	*Hyd_melanos*	0.75	lp	Msurf	0.10	l	Msurf
Hydrophiinae	*Hydrophis*	*Hyd_melanos*	1	lqp	Msurf	0.25	l	Msurf
Hydrophiinae	*Hydrophis*	*Hyd_melanos*	1	lp	Msurf	0.25	lp	Msurf
Hydrophiinae	*Hydrophis*	*Hyd_melanos*	1	l	Msurf	0.50	qp	Msurf
Hydrophiinae	*Hydrophis*	*Hyd_melanos*	2	q	Msurf	0.50	lqp	Msurf
Hydrophiinae	*Hydrophis*	*Hyd_melanos*	2	lq	Msurf	0.50	l	Msurf
Hydrophiinae	*Hydrophis*	*Hyd_melanos*	-	-	-	0.50	lp	Msurf

Hydrophiinae	*Hydrophis*	*Hyd_melanos*	-	-	-	1	lq	Msurf
Hydrophiinae	*Hydrophis*	*Hyd_melanos*	-	-	-	1	q	Msurf
Hydrophiinae	*Hydrophis*	*Hyd_nig*	0.50	p	Bma	0.25	p	Bsurf
Hydrophiinae	*Hydrophis*	*Hyd_nig*	0.75	p	Bma	0.25	lp	Bsurf
Hydrophiinae	*Hydrophis*	*Hyd_nig*	1	lqp	Bma	0.50	lp	Bsurf
Hydrophiinae	*Hydrophis*	*Hyd_nig*	1	qp	Bma	0.50	lqp	Bsurf
Hydrophiinae	*Hydrophis*	*Hyd_nig*	2	lq	Bma	0.75	l	Bsurf
Hydrophiinae	*Hydrophis*	*Hyd_nig*	2	q	Bma	1	l	Bsurf
Hydrophiinae	*Hydrophis*	*Hyd_oce*	0.50	lq	Bsurf	0.10	lp	Bmi
Hydrophiinae	*Hydrophis*	*Hyd_orn*	2	lq	Bmi	0.10	p	Bme
Hydrophiinae	*Hydrophis*	*Hyd_pac*	0.50	l	Bme	0.10	l	Bme
Hydrophiinae	*Hydrophis*	*Hyd_per*	2	l	Bmi	0.10	lq	Msurf
Hydrophiinae	*Hydrophis*	*Hyd_pla*	0.25	qp	Bsurf	0.5	lqp	Bsurf
Hydrophiinae	*Hydrophis*	*Hyd_sch*	0.50	p	Msurf	0.10	p	Msurf
Hydrophiinae	*Hydrophis*	*Hyd_sch*	-	-	-	0.25	p	Msurf
Hydrophiinae	*Hydrophis*	*Hyd_sch*	-	-	-	0.50	p	Msurf
Hydrophiinae	*Hydrophis*	*Hyd_spi*	0.50	qp	Bmi	0.75	p	Bmi
Hydrophiinae	*Hydrophis*	*Hyd_spi*	0.50	lqp	Bmi	0.75	lp	Bmi
Hydrophiinae	*Hydrophis*	*Hyd_spi*	0.75	lqp	Bmi	0.75	lqp	Bmi
Hydrophiinae	*Hydrophis*	*Hyd_spi*	0.75	qp	Bmi	0.75	qp	Bmi
Hydrophiinae	*Hydrophis*	*Hyd_spi*	0.75	p	Bmi	1	p	Bmi

Hydrophiinae	*Hydrophis*	*Hyd_spi*	0.75	lp	Bmi	1	lp	Bmi
Hydrophiinae	*Hydrophis*	*Hyd_spi*	1	lqp	Bmi	1	qp	Bmi
Hydrophiinae	*Hydrophis*	*Hyd_spi*	1	qp	Bmi	1	lqp	Bmi
Hydrophiinae	*Hydrophis*	*Hyd_spi*	1	lp	Bmi	-	-	-
Hydrophiinae	*Hydrophis*	*Hyd_spi*	1	p	Bmi	-	-	-
Hydrophiinae	*Hydrophis*	*Hyd_spi*	2	l	Bmi	-	-	-
Hydrophiinae	*Hydrophis*	*Hyd_spi*	2	p	Bmi	-	-	-
Hydrophiinae	*Hydrophis*	*Hyd_spi*	2	lp	Bmi	-	-	-
Hydrophiinae	*Hydrophis*	*Hyd_sto*	0.10	p	Bsurf	0.10	p	Bsurf
Hydrophiinae	*Hydrophis*	*Hyd_tor*	0.10	p	Bsurf	0.10	lp	Bme
Hydrophiinae	*Hydrophis*	*Hyd_tor*	0.50	p	Bma	0.10	lp	Bmi
Hydrophiinae	*Hydrophis*	*Hyd_tor*	0.50	p	Bme	0.10	p	Bme
Hydrophiinae	*Hydrophis*	*Hyd_tor*	-	-	-	0.10	p	Bmi
Hydrophiinae	*Hydrophis*	*Hyd_tor*	-	-	-	0.10	p	Msurf
Hydrophiinae	*Hydrophis*	*Hyd_tor*	-	-	-	0.25	lp	Bme
Hydrophiinae	*Hydrophis*	*Hyd_tor*	-	-	-	0.25	l	Bmi
Hydrophiinae	*Hydrophis*	*Hyd_tor*	-	-	-	0.25	p	Bme
Hydrophiinae	*Hydrophis*	*Hyd_tor*	-	-	-	0.25	lp	Bmi
Hydrophiinae	*Hydrophis*	*Hyd_tor*	-	-	-	0.25	p	Bmi
Hydrophiinae	*Hydrophis*	*Hyd_tor*	-	-	-	0.50	l	Bme
Hydrophiinae	*Hydrophis*	*Hyd_tor*	-	-	-	0.50	l	Bmi

Hydrophiinae	*Hydrophis*	*Hyd_tor*	-	-	-	0.50	qp	Bme
Hydrophiinae	*Hydrophis*	*Hyd_tor*	-	-	-	0.50	lqp	Bme
Hydrophiinae	*Hydrophis*	*Hyd_tor*	-	-	-	0.50	lp	Bme
Hydrophiinae	*Hydrophis*	*Hyd_tor*	-	-	-	0.75	l	Bme
Hydrophiinae	*Hydrophis*	*Hyd_tor*	-	-	-	0.75	l	Bmi
Hydrophiinae	*Hydrophis*	*Hyd_tor*	-	-	-	1	lq	Bme
Hydrophiinae	*Hydrophis*	*Hyd_vip*	0.25	p	Bme	0.25	p	Bme
Hydrophiinae	*Hydrophis*	*Hyd_vip*	0.50	p	Bmi	0.50	p	Bmi
Hydrophiinae	*Hydrophis*	*Hyd_vip*	0.50	l	Bmi	0.50	l	Bmi
Hydrophiinae	*Hydrophis*	*Hyd_vip*	0.50	p	Bme	0.50	l	Bme
Hydrophiinae	*Hydrophis*	*Hyd_vip*	0.75	p	Bmi	0.75	p	Bmi
Hydrophiinae	*Hydrophis*	*Hyd_vip*	0.75	l	Bmi	0.75	l	Bmi
Hydrophiinae	*Hydrophis*	*Hyd_vip*	1	p	Bmi	1	l	Bmi
Hydrophiinae	*Hydrophis*	*Hyd_vip*	1	l	Bmi	1	p	Bmi
Hydrophiinae	*Hydrophis*	*Hyd_zwe*	0.10	q	Bme	0.10	l	Bme
Hydrophiinae	*Hydrophis*	*Hyd_zwe*	0.25	q	Bme	0.25	l	Bme
Hydrophiinae	*Hydrophis*	*Hyd_zwe*	0.50	p	Bsurf	0.50	l	Bme
Hydrophiinae	*Hydrophis*	*Hyd_zwe*	0.50	q	Bme	0.75	l	Bma
Hydrophiinae	*Hydrophis*	*Hyd_zwe*	0.75	p	Bsurf	2	l	Bma
Hydrophiinae	*Hydrophis*	*Hyd_zwe*	0.75	l	Bme	2	lq	Bma
Hydrophiinae	*Hydrophis*	*Hyd_zwe*	3	lq	Bma	3	l	Bma

Hydrophiinae	*Hydrophis*	*Hyd_zwe*	3	l	Bma	3	lq	Bma
Hydrophiinae	*Hydrophis*	*Hyd_zwe*	4	lqp	Bma	4	l	Bma
Hydrophiinae	*Hydrophis*	*Hyd_zwe*	4	lp	Bma	4	lq	Bma
Hydrophiinae	*Hydrophis*	*Hyd_zwe*	4	l	Bma	-	-	-
Hydrophiinae	*Hydrophis*	*Hyd_zwe*	4	lq	Bma	-	-	-
Laticaudinae	*Laticauda*	*Lat_col*	0.10	qp	Bmi	0.75	q	Bma
Laticaudinae	*Laticauda*	*Lat_cro*	3	p	Msurf	0.10	lqp	Bmi
Laticaudinae	*Laticauda*	*Lat_cro*	-	-	-	0.10	l	Bmi
Laticaudinae	*Laticauda*	*Lat_cro*	-	-	-	0.10	lp	Bmi
Laticaudinae	*Laticauda*	*Lat_cro*	-	-	-	0.10	q	Bmi
Laticaudinae	*Laticauda*	*Lat_cro*	-	-	-	0.25	lq	Bmi
Laticaudinae	*Laticauda*	*Lat_cro*	-	-	-	0.25	l	Bmi
Laticaudinae	*Laticauda*	*Lat_cro*	-	-	-	0.25	lqp	Bmi
Laticaudinae	*Laticauda*	*Lat_cro*	-	-	-	0.25	lp	Bmi
Laticaudinae	*Laticauda*	*Lat_cro*	-	-	-	0.50	lq	Bmi
Laticaudinae	*Laticauda*	*Lat_cro*	-	-	-	0.50	l	Bmi
Laticaudinae	*Laticauda*	*Lat_cro*	-	-	-	0.75	lq	Bmi
Laticaudinae	*Laticauda*	*Lat_cro*	-	-	-	0.75	l	Bmi
Laticaudinae	*Laticauda*	*Lat_fro*	0.10	lqp	Bmi	0.10	l	Bmi
Laticaudinae	*Laticauda*	*Lat_fro*	0.10	lp	Bmi	0.10	lq	Bmi
Laticaudinae	*Laticauda*	*Lat_fro*	0.10	lq	Bmi	0.25	l	Bmi

Laticaudinae	*Laticauda*	*Lat_fro*	0.10	l	Bmi	0.25	lq	Bmi
Laticaudinae	*Laticauda*	*Lat_fro*	0.10	q	Bmi	0.25	lp	Bmi
Laticaudinae	*Laticauda*	*Lat_fro*	0.25	lqp	Bmi	0.25	lqp	Bmi
Laticaudinae	*Laticauda*	*Lat_fro*	0.25	lp	Bmi	0.50	l	Bmi
Laticaudinae	*Laticauda*	*Lat_fro*	0.25	qp	Bmi	0.50	lq	Bmi
Laticaudinae	*Laticauda*	*Lat_fro*	0.25	lq	Bmi	0.50	lp	Bmi
Laticaudinae	*Laticauda*	*Lat_fro*	0.25	l	Bmi	0.50	lqp	Bmi
Laticaudinae	*Laticauda*	*Lat_fro*	0.25	q	Bmi	0.75	l	Bmi
Laticaudinae	*Laticauda*	*Lat_fro*	0.50	lq	Bmi	0.75	lq	Bmi
Laticaudinae	*Laticauda*	*Lat_fro*	0.50	l	Bmi	0.75	lp	Bmi
Laticaudinae	*Laticauda*	*Lat_fro*	0.50	lp	Bmi	0.75	lqp	Bmi
Laticaudinae	*Laticauda*	*Lat_fro*	0.50	lqp	Bmi	1	l	Bmi
Laticaudinae	*Laticauda*	*Lat_fro*	0.50	q	Bmi	1	lq	Bmi
Laticaudinae	*Laticauda*	*Lat_fro*	0.75	lq	Bmi	-	-	-
Laticaudinae	*Laticauda*	*Lat_fro*	0.75	l	Bmi	-	-	-
Laticaudinae	*Laticauda*	*Lat_fro*	1	lq	Bmi	-	-	-
Laticaudinae	*Laticauda*	*Lat_fro*	1	l	Bmi	-	-	-
Laticaudinae	*Laticauda*	*Lat_lat*	0.10	qp	Bme	3	lp	Bme
Laticaudinae	*Laticauda*	*Lat_lat*	0.25	qp	Bme	3	p	Bme
Laticaudinae	*Laticauda*	*Lat_lat*	-	-	-	4	lp	Bme
Laticaudinae	*Laticauda*	*Lat_sai*	0.10	lq	Bme	0.10	l	Bmi

Laticaudinae	*Laticauda*	*Lat_sai*	0.25	lq	Bme	0.10	lqp	Bmi
Laticaudinae	*Laticauda*	*Lat_sai*	0.75	lq	Bme	0.25	lq	Bmi
Laticaudinae	*Laticauda*	*Lat_sai*	-	-	-	0.25	l	Bmi
Laticaudinae	*Laticauda*	*Lat_sai*	-	-	-	0.50	lq	Bmi
Laticaudinae	*Laticauda*	*Lat_sai*	-	-	-	0.50	l	Bmi
Laticaudinae	*Laticauda*	*Lat_sai*	-	-	-	0.75	l	Bmi
Laticaudinae	*Laticauda*	*Lat_sai*	-	-	-	1	lq	Bmi
Laticaudinae	*Laticauda*	*Lat_sch*	0.50	p	Bmi	0.10	lq	Bme
Laticaudinae	*Laticauda*	*Lat_sch*	0.75	p	Bmi	-	-	-
Laticaudinae	*Laticauda*	*Lat_sch*	1	p	Bmi	-	-	-
Laticaudinae	*Laticauda*	*Lat_sem*	4	q	Bsurf	0.25	lq	Bma
Laticaudinae	*Laticauda*	*Lat_sem*	-	-	-	0.25	lqp	Bma
Laticaudinae	*Laticauda*	*Lat_sem*	-	-	-	0.25	qp	Bma
