# Supplementary material for: Global analysis of the influence of environmental variables to explain ecological niches and realized thermal niche boundaries of sea snakes
Source: PLoS One. 2024 Dec 5;19(12):e0310456. doi: 10.1371/journal.pone.0310456 (PMC11620380; doi:10.1371/journal.pone.0310456)
Supplement: S3 Table — This table contains the results of the non-parametric Wilcoxon test of MaxEnt metric across taxa level and spatial resolution. (PDF) [file pone.0310456.s003.pdf]

S3A Table. Non-parametric Wilcoxon Test comparing the Percentage Contribution (PC)and Permutation Importance (PI) metrics calculated by MaxEnt at 5 arc-minute resolution, across lineage, family, and genus levels. Cal = Calcite, Cvel = Currents velocity, Doxy = Dissolved molecular oxygen, Iro = Iron, Nit = Nitrates, pH = pH, Pho = Phosphates, Sal = Salinity, Sil = Silicates, Tem = Temperature, * = medians statistically different ($p < 0.05$).

	**Cal**	**Cvel**	**Doxy**	**Iro**	**Nit**	**pH**	**Pho**	**Sal**	**Sil**	**Tem**
Sea snakes	0.09	1.00	0.92	0.86	0.35	0.73	0.80	< 0.05*	0.19	0.53
Hydrophiinae	0.09	0.82	0.69	0.95	0.44	0.73	0.91	< 0.05*	0.31	0.51
Laticaudinae	NA	0.69	0.20	0.84	1.00	NA	1.00	0.70	0.70	0.84
Aipysurus	0.33	0.94	0.69	0.59	1.00	NA	1.00	0.21	NA	0.88
Hydrophis	0.69	0.58	0.46	0.90	0.39	0.89	0.96	< 0.05*	0.49	0.33
Emydocephalus	NA	1.00	1.00	0.67	NA	NA	NA	1.00	1.00	1.00
Laticauda	NA	0.69	0.20	0.84	1.00	NA	1.00	0.70	0.70	0.84

S3B Table. Non-parametric Wilcoxon Test comparing the Percentage Contribution (PC) and Permutation Importance (PI) metrics calculated by MaxEnt at 10 arc-minute resolution, across lineage, family, and genus levels. Cal = Calcite, Cvel = Currents velocity, Doxy = Dissolved molecular oxygen, Iro = Iron, Nit = Nitrates, pH = pH, Pho = Phosphates, Sal = Salinity, Sil = Silicates, Tem = Temperature, * = medians statistically different ($p < 0.05$).

	**Cal**	**Cvel**	**Doxy**	**Iro**	**Nit**	**pH**	**Pho**	**Sal**	**Sil**	**Tem**
Sea snakes	0.22	0.38	0.87	0.99	0.17	0.71	0.54	< 0.05*	0.71	0.74
Hydrophiinae	0.22	0.57	0.80	0.91	0.17	0.71	0.54	< 0.05*	0.59	0.94
Laticaudinae	NA	0.41	1.00	1.00	NA	NA	NA	1.00	1.00	0.32
Aipysurus	0.10	0.71	1.00	1.00	1.00	1.00	1.00	0.21	1.00	0.80
Emydocephalus	1.00	1.00	NA	1.00	NA	NA	NA	1.00	1.00	0.67
Hydrophis	0.59	0.75	0.90	0.95	0.15	0.69	0.66	< 0.05*	0.34	0.99
Laticauda	NA	0.41	1.00	1.00	NA	NA	NA	1.00	1.00	0.32

S3C Table. Non-parametric Wilcoxon Test comparing the Percentage Contribution (PC) metric calculated by MaxEnt across lineage, family, and genus levels at 5 and 10 arc-minute resolutions. Cal = Calcite, Cvel = Currents velocity, Doxy = Dissolved molecular oxygen, Iro = Iron, Nit = Nitrates, pH = pH, Pho = Phosphates, Sal = Salinity, Sil = Silicates, Tem = Temperature, * = medians statistically different ($p < 0.05$).

	**Cal**	**Cvel**	**Doxy**	**Iro**	**Nit**	**pH**	**Pho**	**Sal**	**Sil**	**Tem**
Sea snakes	0.64	0.11	0.92	0.44	1.00	0.43	0.86	0.48	0.02*	0.97
Hydrophiinae	0.64	0.16	0.88	0.54	1.00	0.43	0.96	0.60	0.05	0.33
Laticaudinae	NA	0.53	0.80	0.57	NA	NA	NA	0.80	0.50	0.05
Aipysurus	0.20	0.45	0.80	0.53	0.80	0.67	1.00	1.00	NA	0.37
Hydrophis	0.61	0.35	0.66	0.97	0.82	0.90	0.94	0.73	0.11	0.83
Emydocephalus	NA	1.00	NA	1.00	NA	NA	NA	1.00	1.00	0.67
Laticauda	NA	0.53	0.80	0.57	NA	NA	NA	0.80	0.50	0.05

S3D Table. Non-parametric Wilcoxon Test comparing the Permutation Importance (PI) metric calculated by MaxEnt across lineage, family, and genus levels at 5 and 10 arc-minute resolutions. Cal = Calcite, Cvel = Currents velocity, Doxy = Dissolved molecular oxygen, Iro = Iron, Nit = Nitrates, pH = pH, Pho = Phosphates, Sal = Salinity, Sil = Silicates, Tem = Temperature, * = medians statistically different ($p < 0.05$).

	**Cal**	**Cvel**	**Doxy**	**Iro**	**Nit**	**pH**	**Pho**	**Sal**	**Sil**	**Tem**
Sea snakes	1.00	0.33	0.54	0.70	0.98	0.79	0.84	0.49	0.12	0.82
Hydrophiinae	1.00	0.38	0.73	0.57	0.86	0.79	1.00	0.34	0.25	0.23
Laticaudinae	NA	0.65	1.00	0.86	NA	NA	NA	0.80	0.50	0.03*
Aipysurus	0.80	0.45	0.80	0.53	0.40	NA	0.67	0.61	NA	0.67
Hydrophis	0.76	0.78	0.66	0.91	0.76	0.73	0.66	0.59	0.11	0.31
Emydocephalus	NA	1.00	NA	1.00	NA	NA	NA	1.00	1.00	1.00
Laticauda	NA	0.65	1.00	0.86	NA	NA	NA	0.80	0.50	0.03*
