# Supplementary material for: Global analysis of the influence of environmental variables to explain ecological niches and realized thermal niche boundaries of sea snakes
Source: PLoS One. 2024 Dec 5;19(12):e0310456. doi: 10.1371/journal.pone.0310456 (PMC11620380; doi:10.1371/journal.pone.0310456)
Supplement: S4 Table — This table contains the results of the homogeneity of variances of thermal limits by the non-parametric Fligner-Killeen test. (PDF). (PDF) [file pone.0310456.s004.pdf]

S4A Table. Summary of *p* values for homogeneity of variances of lower and upper realized thermal limits by the minimum, mean, and maximum temperatures across lineage, family, and genus levels at 5 arcmin by non-parametric Fligner-Killeen test. Genus *Laticauda* were excluded for the analysis due to the represent the same result for the Laticaudinae subfamily. Tem = temperature, Max = maximum, Min = minimum, Bma = Bio-Oracle benthic maximum depth, Bme = Bio-Oracle benthic mean depth, Bmi = Bio-Oracle benthic minimum depth, Bsurf = Bio-Oracle surface, Msurf = MARPEC surface, * = statistically different variances ($p > 0.05$).

	**Tem**	**Bma**	**Bme**	**Bmi**	**Bsurf**	**Msurf**
Sea snakes	Max	3.9e-6*	4.3e-11*	1.5e-14*	8.0e-5*	1.5e-4*
Sea snakes	Mean	5.8e-6*	1.4e-12*	3.9e-15*	4.6e-8*	7.9e-10*
Sea snakes	Min	3.1e-3*	3.2e-09*	2.9e-13*	1.4e-3*	8.9e-6*
Hydrophiinae	Max	1.3e-7*	3.1e-11*	2.2e-12*	4.7e-5*	2.7e-4*
Hydrophiinae	Mean	1.7e-7*	4.0e-11*	1.7e-12*	1.9e-8*	1.7e-9*
Hydrophiinae	Min	5.3e-5*	1.3e-09*	1.5e-11*	4.2e-3*	4.3e-6*
Laticaudinae	Max	0.28	0.42	1.8e-2*	0.31	0.11
Laticaudinae	Mean	0.1	0.52	1.8e-2*	0.19	0.53
Laticaudinae	Min	0.07	0.8	1.8e-2*	4.8e-2*	0.08
Aipysurus	Max	2.0e-2*	2.3e-2*	1.6e-2*	2.8e-2*	4.0e-2*
Aipysurus	Mean	2.26e-2*	1.61e-2*	3.07e-2*	0.3	0.15
Aipysurus	Min	2.81e-2*	2.16e-2*	3.89e-2*	0.3	0.2
Emydocephalus	Max	0.12	0.12	0.12	0.13	0.13
Emydocephalus	Mean	0.08	0.08	0.08	0.08	0.12
Emydocephalus	Min	0.1	0.12	0.08	0.08	0.12
Hydrophis	Max	2.9e-6*	8.6e-7*	2.1e-6*	3.5e-3*	6.7e-3*
Hydrophis	Mean	1.8e-6*	1.3e-7*	1.5e-7*	2.6e-8*	1.8e-8*
Hydrophis	Min	1.7e-4*	5.7e-6*	9.1e-6*	1.4e-3*	3.6e-6*

S4B Table. Summary of *p* values for homogeneity of variances of lower and upper realized thermal limits by the minimum, mean, and maximum temperatures across lineage, family, and genus levels at 10 arcmin by non-parametric Fligner-Killeen test. Genus *Laticauda* were excluded for the analysis due to the represent the same result for the Laticaudinae subfamily. Tem = temperature, Max = maximum, Min = minimum, Bma = Bio-Oracle benthic maximum depth, Bme = Bio-Oracle benthic mean depth, Bmi = Bio-Oracle benthic minimum depth, Bsurf = Bio-Oracle surface, Msurf = MARPEC surface, * = statistically different variances (p > 0.05).

	**Tem**	**Bma**	**Bme**	**Bmi**	**Bsurf**	**Msurf**
Sea snakes	Max	4.3e-7*	2.9e-11*	1.0e-13*	1.7e-4*	2.2e-4*
Sea snakes	Mean	1.5e-6*	5.2e-11*	1.1e-13*	4.2e-8*	4.3e-10*
Sea snakes	Min	2.0e-4*	1.5e-9*	1.6e-12*	6.9e-4*	9.8e-6*
Hydrophiinae	Max	3.1e-10*	8.5e-12*	2.6e-12*	6.9e-5*	1.4e-4*
Hydrophiinae	Mean	1.4e-9*	1.1e-11*	1.6e-12*	2.5e-8*	1.9e-9*
Hydrophiinae	Min	1.8e-6*	8.0e-11*	3.4e-11*	2.3e-3*	1.3e-5*
Laticaudinae	Max	0.42	0.6	2.4e-2*	0.31	0.18
Laticaudinae	Mean	0.07	0.81	2.4e-2*	0.2	0.25
Laticaudinae	Min	4.4e-2*	0.77	2.4e-2*	4.4e-2*	0.08
Aipysurus	Max	2.0e-2*	5.2e-3*	8.0e-3*	2.2e-2*	4.5e-2*
Aipysurus	Mean	2.81e-2*	1.12e-02*	1.6e-2*	0.3	0.14
Aipysurus	Min	2.8e-2*	9.8e-3*	1.4e-2*	0.23	0.23
Emydocephalus	Max	0.08	0.08	0.08	0.08	0.1
Emydocephalus	Mean	0.08	0.08	0.08	0.08	0.12
Emydocephalus	Min	0.08	0.08	0.08	0.08	0.12
Hydrophis	Max	1.9e-6*	1.3e-6*	3.9e-6*	3.5e-3*	4.5e-3*
Hydrophis	Mean	2.2e-6*	2.5e-7*	4.1e-7*	5.4e-8*	2.3e-8*
Hydrophis	Min	2.8e-4*	1.8e-5*	2.7e-5*	2.1e-3*	1.7e-5*

S4C Table. Summary of statistical analyses and *p*-values comparing the minimum, mean, and maximum temperatures for both lower and upper realized thermal limits using the Shapiro-Wilk test across lineage, family, and genus levels at 5 and 10 arcmin. * = a *p*-value <0.05 indicating a departure from normal distribution.

			5 arcmin		10 arcmin	
	**Limit**	**Temperature**	**W**	***p*-value**	**W**	***p*-value**
Sea snakes	Lower	Minimum	0.90	8.46e-12*	0.90	1.05e-11*
Sea snakes	Lower	Mean	0.84	2.76e-15*	0.86	3.05e-14*
Sea snakes	Lower	Maximum	0.81	1.27e-16*	0.83	9.23e-16*
Sea snakes	Upper	Minimum	0.61	2.19e-23*	0.67	9.13e-22*
Sea snakes	Upper	Mean	0.37	1.76e-28*	0.43	1.79e-27*
Sea snakes	Upper	Maximum	0.47	1.12e-26*	0.52	1.27e-25*
Hydrophiinae	Lower	Minimum	0.90	3.55e-11*	0.90	3.65e-11*
Hydrophiinae	Lower	Mean	0.84	2.67e-14*	0.83	2.34e-13*
Hydrophiinae	Lower	Maximum	0.81	1.42e-15*	0.86	7.40e-15*
Hydrophiinae	Upper	Minimum	0.57	4.76e-23*	0.67	1.36e-20*
Hydrophiinae	Upper	Mean	0.33	1.09e-27*	0.40	1.87e-26*
Hydrophiinae	Upper	Maximum	0.42	3.85e-26*	0.50	1.33e-24*
Laticaudinae	Lower	Minimum	0.86	1.19e-03*	0.88	2.23e-03*
Laticaudinae	Lower	Mean	0.82	1.78e-04*	0.85	5.66e-04*
Laticaudinae	Lower	Maximum	0.80	8.06e-05*	0.83	3.01e-04*
Laticaudinae	Upper	Minimum	0.82	1.28e-04*	0.71	1.86e-06*
Laticaudinae	Upper	Mean	0.70	1.70e-06*	0.55	2.12e-08*
Laticaudinae	Upper	Maximum	0.69	1.01e-06*	0.48	3.44e-09*
Aipysurus	Lower	Minimum	0.88	2.77e-04*	0.86	6.41e-05*
Aipysurus	Lower	Mean	0.79	1.53e-06*	0.82	7.75e-06*
Aipysurus	Lower	Maximum	0.81	4.30e-06*	0.81	4.72e-06*
Aipysurus	Upper	Minimum	0.84	2.56e-05*	0.86	8.21e-05*
Aipysurus	Upper	Mean	0.68	1.32e-08*	0.70	2.36e-08*
Aipysurus	Upper	Maximum	0.68	1.49e-08*	0.71	3.36e-08*

Emydocephalus	Lower	Minimum	0.9	0.25	0.96	0.75
Emydocephalus	Lower	Mean	0.89	0.17	0.94	0.58
Emydocephalus	Lower	Maximum	0.89	0.17	0.94	0.53
Emydocephalus	Upper	Minimum	0.90	0.20	0.93	0.46
Emydocephalus	Upper	Mean	0.85	0.06	0.90	0.22
Emydocephalus	Upper	Maximum	0.85	0.06	0.93	0.47
Hydrophis	Lower	Minimum	0.89	1.15e-09*	0.89	8.77e-10*
Hydrophis	Lower	Mean	0.84	6.25e-12*	0.85	1.40e-11*
Hydrophis	Lower	Maximum	0.81	2.67e-13*	0.82	6.94e-13*
Hydrophis	Upper	Minimum	0.91	2.65e-08*	0.93	1.11e-06*
Hydrophis	Upper	Mean	0.96	2.70e-04*	0.97	5.03e-04*
Hydrophis	Upper	Maximum	0.88	4.42e-10*	0.90	4.13e-09*
