# Supplementary material for: Global analysis of the influence of environmental variables to explain ecological niches and realized thermal niche boundaries of sea snakes
Source: PLoS One. 2024 Dec 5;19(12):e0310456. doi: 10.1371/journal.pone.0310456 (PMC11620380; doi:10.1371/journal.pone.0310456)
Supplement: S1 File — These figures show the contribution of each species estimated by MaxEnt metrics at both resolutions. (PDF) [file pone.0310456.s005.pdf]

Figure SM1. All variable contribution estimated by MaxEnt percentage of contribution (PC) and permutation of importance (PI) for each species at 5 arc-minutes. Warmer colors denoted higher contribution while lighter colors less contribution.

	PC										PI									
	Cal	Cvel	Doxy	Iro	Nit	pH	Pho	Sal	Sil	Tem	Cal	Cvel	Doxy	Iro	Nit	pH	Pho	Sal	Sil	Tem
Aip_apr	0	81.16	0	3.76	0	0	0	0	0	16.58	0	81.63	0	1.85	0	0	0	0	0	14.36
Aip_dub	0	3.34	19.96	0.23	0	0	0	3.77	0	72.64	0	0.89	64.99	0.62	0	0	0	0.26	0	31.95
Aip_eyd	20.74	0.02	0.03	52.85	7.03	0.21	4.34	6.39	0	8.39	0.36	0.22	2.34	82.54	6.26	0	0.84	1.24	0	6.19
Aip_fol	0	0	0	0	0	0	0	100	0	0	0	0	0	0	0	0	0	100	0	0
Aip_fus	0	7.12	10.44	31.86	0	0	0	0.37	0	50.21	0	4.36	12.6	46.47	0	0	0	0.15	0	36.42
Aip_lae	0	0.23	48.09	1.22	0	0	0	2.26	0	48.21	0	0.3	38.99	3.65	0	0	0	0.04	0	57.01
Aip_mos	13.58	8.83	0	41.53	4.77	0	0	26.82	0	4.47	3.58	5.09	0	73.16	4.8	0	0	0.45	0	12.92
Aip_poo	0	0	0	0	0	0	0	70.25	0	10.34	0	0	0	0	0	0	0	61.11	0	17.39
Aip_ten	0	0	0	0	0	0	57.01	30.37	0	14.6	0	0	0	0	0	0	61.32	11	0	22.21
Emy_ann	0	5.4	39.81	5.95	0	0	0	0	23.35	25.49	0	4.43	2.23	5.28	0	0	0	0	79.11	8.94
Emy_iji	0	0	0	0.56	0	0	0	47.58	0	51.29	0	0	0	0.09	0	0	0	0.09	0	99.82
Hyd_atr	0	1.67	2.32	0.21	0	0	0	5.12	0	87.96	0	1.07	6.95	0.82	0	0	0	3.99	0	86.56
Hyd_bel	0	3.74	0	2.09	0	0	0	0	0	93.86	0	4.71	0	4.44	0	0	0	0	0	90.91
Hyd_bro	0	0.03	0	0.95	0	0	0.18	0	1.87	96.97	0	0.32	0	0.27	0	0	70.86	0	1.6	26.95
Hyd_cae	0	8.24	0	39.71	15.03	0	0	0.97	0	36.84	0	1.94	0	22.89	65.16	0	0	0.46	0	9.1
Hyd_cog	0	0	0	1.75	0	0	0	57.45	0	41.17	0	0	0	0.68	0	0	0	18.83	0	80.17
Hyd_cur	0	3.26	5.63	5.98	0	0	16.51	30.42	29.43	8.77	0	2.88	4.87	7.97	0	0	4.13	0.62	72.19	7.33
Hyd_cya	0	7.99	0	9.3	0	0	0	13.99	0	67.95	0	6.76	0	7.62	0	0	0	1	0	85.57
Hyd_cze	0	0	0	0	0	0	0	34.08	0	65.92	0	0	0	0	0	0	0	36.78	0	63.22
Hyd_ele	0	2.04	42.28	5.47	0	0	0	1.91	4.44	43.87	0	0.88	15.26	4.72	0	0	0	0.24	29.65	49.26
Hyd_fas	0	2.63	1.02	2.62	0	0	0	26.89	1.67	62.07	0	6.98	0.62	5.18	0	0	0	0.2	12.81	66.84
Hyd_gra	0	0.31	0.77	0	0	0	0	48.82	0	49.7	0	1.56	2.54	0	0	0	0	4.59	0	87.93
Hyd_har	0	0	0	0	0	0	0	3.17	0	96.83	0	0	0	0	0	0	0	0.12	0	99.88
Hyd_ino	0	10.83	0	8.43	0	0	0	36.43	0	41.89	0	5.55	0	8.43	0	0	0	1.18	0	81.11
Hyd_jer	0	73.49	0	0	0	0	26.51	0	0	0	0	45.31	0	0	0	0	54.69	0	0	0
Hyd_kin	0	0	0	0	0	0	0	61.32	0	38.68	0	0	0	0	0	0	0	32.61	0	67.39
Hyd_klo	0	0	50	0	0	0	0	0	0	50	0	0	28.7	0	0	0	0	0	0	71.3
Hyd_lap	0.44	0.02	0.01	49.55	0	0.45	34.04	0	0	15.51	4.41	0	0	59.62	0	30.52	4.88	0	0	0.57
Hyd_mac	0	1.71	0	19.37	64.53	0	0	0	0	14.39	0	11.11	0	18.53	64.75	0	0	0	0	5.61
Hyd_maj	0	3.36	0	13.88	69.08	0	0	0	0	13.68	0	2.18	0	8.04	48.97	0	0	0	0	40.81
Hyd_melanoc	0	17.78	0	57.03	0	0	0	0	0	25.19	0	30.93	0	59.63	0	0	0	0	0	9.44
Hyd_melanos	0	0	0	0	0	0	0	65.21	0	34.79	0	0	0	0	0	0	0	45.86	0	54.14
Hyd_nig	0	0.42	0	14.71	0	0	0	42.89	0	42.4	0	1.02	0	7.14	0	0	0	0.32	0	91.35
Hyd_oce	11.28	1.63	0	44.63	11.1	3.8	11.98	0	0	15.59	4.54	2.18	0	44.28	29.91	0.12	2.93	0	0	16.04
Hyd_orn	0	0.06	0	3.67	6.97	0	50.38	1.61	0	37.31	0	0.97	0	9.14	79.09	0	2.82	4.12	0	3.87
Hyd_pac	0	5.28	0.3	4.43	39.02	0	0	5.56	0	45.42	0	3.09	4.2	0.87	29.32	0	0	5.45	0	57.07
Hyd_per	0	0.31	0	6.27	88.12	0	1.15	0	0	4.15	0	0.07	0	6.67	70.1	0	12.62	0	0	10.55
Hyd_pla	4.04	5.71	0	19.23	4.05	10.95	22.23	14.69	0	19.09	0.79	3.11	0	20.42	12.49	6.96	21.65	12.54	0	22.05
Hyd_sch	0	0	0	0	0	0	0	42.92	0	57.08	0	0	0	0	0	0	0	9.11	0	90.89
Hyd_spi	0	2.61	0	1.35	0	0	0	17.68	0	76.55	0	5.99	0	1.83	0	0	0	3.35	0	88.03
Hyd_sto	9.22	4.3	0	40.91	8.59	4.95	0	25.23	0	6.79	1.75	3.39	0	60.79	22.39	4.62	0	4.46	0	2.59
Hyd_tor	0	1.81	0	47.61	0	0	0	0	0	49.82	0	7.84	0	25.08	0	0	0	0	0	63.49
Hyd_vip	0	6.73	0	22.06	0	0	0	25.49	0	44.52	0	15.79	0	7.56	0	0	0	1.31	0	74.08
Hyd_zwe	0	1.7	0	0.04	75.38	0	0	0	0	6.77	0	1.37	0	0.01	43.94	0	0	0	0	0
Lat_col	0	0.87	4.14	5.71	0	0	0	41.86	3.78	43.64	0	0.22	3.37	4.02	0	0	0	0.66	59.81	31.91
Lat_cro	0	0	0	0	0	0	0	96.72	0	3.28	0	0	0	0	0	0	0	88.43	0	11.57
Lat_fro	0	0	0	0	77.31	0	0	0	0	22.69	0	0	0	0	69.59	0	0	0	0	30.41
Lat_lat	0	3.45	9.17	10.71	0	0	0	0	59.12	17.54	0	0.88	6.57	8.93	0	0	0	0	56.15	27.47
Lat_sai	0	4.59	0	14.21	0	0	0	3.32	0	78.62	0	2.47	0	20.78	0	0	0	7.36	0	73.5
Lat_sch	0	0	47.89	30.28	1.53	0	0	0	0.52	19.78	0	0	0.35	47.77	49.66	0	0	0	1.42	0.8
Lat_sem	0	27.06	0	0.8	0	0	67.35	0	0	4.8	0	58.8	0	8.19	0	0	33.01	0	0	0

Figure SM2. All variable contribution estimated by MaxEnt percentage of contribution (PC) and permutation of importance (PI) for each species at 10 arc-minutes. Warmer colors denoted higher contribution while lighter colors less contribution.

	PC										PI									
	Cal	Cvel	Doxy	Iro	Nit	pH	Pho	Sal	Sil	Tem	Cal	Cvel	Doxy	Iro	Nit	pH	Pho	Sal	Sil	Tem
Aip_apr	0	62.96	0	10.33	0	0	0	6.07	0	20.68	0	80.21	0	5.78	0	0	0	3.41	0	9.79
Aip_dub	0	1.78	48.24	1.76	0	0	0	4.81	0	43.41	0	1.11	39.93	6.45	0	0	0	0.09	0	52.43
Aip_eyd	10.61	2.31	6.72	35.74	4.18	1.19	14.9	12.97	0	11.38	0.4	1.25	26.19	53.5	6.04	1.78	7.25	1.23	0	2.36
Aip_fol	0	55.06	0	15.74	0	0	0	0	0	29.2	0	63.67	0	18.25	0	0	0	0	0	18.08
Aip_fus	0	10.55	0	47.58	0	0	0	11.91	0	27.45	0	7.78	0	46.69	0	0	0	11.3	0	36.01
Aip_lae	1.85	4.87	0	19.44	12.67	49.57	0	7.81	0	3.79	0.02	2.85	0	6.59	18.24	27.27	0	30.33	0	14.69
Aip_mos	4.56	9.92	0	53.09	11.76	0	0	17.61	0	3.06	0.48	3.43	0	85.09	8.29	0	0	2.44	0	0.27
Aip_poo	0	0	0	0	0	0	0	0	93.62	6.38	0	0	0	0	0	0	0	0	63.7	36.3
Aip_ten	0	0	0	0	0	0	43.99	46.52	0	8.8	0	0	0	0	0	0	62.59	17.32	0	19.06
Emy_ann	0	2.27	0	4.96	0	0	0	33.6	52.95	6.22	0	2.79	0	4.58	0	0	0	4.58	66.63	22.23
Emy_iji	4.27	55.31	0	0	0	0	0	0	0	35.64	10.86	50.96	0	0	0	0	0	0	0	39.4
Hyd_atr	0	1.41	17.36	1.27	0	0	0	1.93	0	77.27	0	1.93	17.09	1.42	0	0	0	1.77	0	75.42
Hyd_bel	0	3.53	5.52	0	0	0	0	5.22	70.04	15.69	0	2.15	10.26	0	0	0	0	0.21	83.91	3.47
Hyd_bro	0	0.72	15.82	0	0	0	0	6.21	0	69.54	0	1.78	10.62	0	0	0	0	0.04	0	87.6
Hyd_cae	0	11.04	0	33.51	15.4	0	0	1.8	0	38.67	0	4.84	0	13.95	66.06	0	0	0.69	0	11.8
Hyd_cog	6.28	2.4	0	3.67	32.7	0.4	39.66	0	0	9.61	14.8	0	0	16.89	49.53	2.98	3.5	0	0	5.36
Hyd_cur	0	0	0	0	0	0	0	13.25	0	86.75	0	0	0	0	0	0	0	3.24	0	96.76
Hyd_cya	0	0	0	0	0	0	0	8.08	0	91.92	0	0	0	0	0	0	0	14.5	0	85.5
Hyd_cze	0	0	0	0	0	0	0	90.68	0	9.32	0	0	0	0	0	0	0	93.02	0	6.98
Hyd_ele	0	2.1	42.08	4.69	0	0	0	2.2	5.14	43.78	0	1.34	14.16	4.02	0	0	0	0.26	42.32	37.92
Hyd_fas	0	3.36	1.33	7.47	0	0	0	7.74	0	78.01	0	4.53	6.56	7.84	0	0	0	1.27	0	79.44
Hyd_gra	0	1.68	0	7	0	0	0	15.41	0	74.44	0	2.31	0	11.5	0	0	0	9.16	0	75.03
Hyd_har	0	0	0	0	0	0	0	33.92	0	66.08	0	0	0	0	0	0	0	67.42	0	32.58
Hyd_ino	0	13.11	0	2.91	0	0	0	38.2	0	42.29	0	14.88	0	6.09	0	0	0	1.68	0	74.02
Hyd_jer	45.01	0	0	0	0	14.12	0	0	0	40.87	55.95	0	0	0	0	44.05	0	0	0	0
Hyd_kin	0	0	0	0	0	0	0	50.07	0	49.93	0	0	0	0	0	0	0	16.49	0	83.51
Hyd_klo	4.28	0	13.34	24.27	0.01	0	0	0	0	49.99	2.91	0	14.68	0.15	0	0	0	0	0	58.65
Hyd_lap	11.2	0.26	0.62	40.02	0	2.26	24.8	0	0	20.83	3.34	0	0.01	72.92	0	8.97	14.76	0	0	0
Hyd_mac	0	2.99	0	23.6	57.77	0	0	0	0	15.63	0	8.66	0	15.62	71.24	0	0	0	0	4.49
Hyd_maj	0	5.4	1.85	5.82	35.3	0	1.23	34.97	5.89	9.53	0	1.04	2.04	4.49	44.52	0	4.41	0.15	37.7	5.65
Hyd_melanoc	0	17.08	0	41.14	0	0	0	0	0	41.78	0	3.01	0	46.1	0	0	0	0	0	50.89
Hyd_melanos	0	0	0	0	0	0	0	54.71	0	45.29	0	0	0	0	0	0	0	35.24	0	64.76
Hyd_nig	0	0	0	43	17.35	0	0	0	0	38.68	0	0	0	58.09	16.95	0	0	0	0	21.23
Hyd_oce	0	2.61	0	3.34	75.16	0	1.56	0	0	17.34	0	4.83	0	4.88	64.24	0	11.97	0	0	14.08
Hyd_orn	0	1.6	1.6	0.35	12.05	0	0	46.94	0	37.46	0	3.17	5.66	1.24	78.85	0	2.02	0	0	9.07
Hyd_pac	0	4.14	0.15	1.23	61.24	0	0	9.86	0	23.39	0	2.21	8.17	3.1	56.23	0	0	6.24	0	24.06
Hyd_per	0	0	0	0	0	0	0	10.94	0	89.06	0	0	0	0	0	0	0	26.07	0	73.93
Hyd_pla	2.01	5.11	0	19.98	5.05	9.67	24.67	16.4	0	17.11	0.42	2.89	0	19.53	10.95	6.18	21.93	16.07	0	22.04
Hyd_sch	0	0	0	0	0	0	0	50	0	50	0	0	0	0	0	0	0	22.22	0	77.78
Hyd_spi	0	3.48	29.68	0.92	0	0	0	16.55	47.66	1.58	0	2.09	5.87	0.93	0	0	0	1.23	84.9	0.81
Hyd_sto	10.54	3.75	0	39.41	8.05	6.08	0	25.72	0	6.45	2.02	2.77	0	60.88	18.37	4.98	0	6.74	0	4.25
Hyd_tor	0	10.02	0	42.97	0	0	0	0	0	49.37	0	13.95	0	29.26	0	0	0	0	0	59.28
Hyd_vip	0	11.69	0	14.62	0	0	0	17.37	0	43.94	0	11.21	0	3.6	0	0	0	0.49	0	83.61
Hyd_zwe	0	0	0	2.11	83.97	0	0	0	0	6.17	0	0	0	5.21	75.76	0	0	0	0	16.11
Lat_col	0	2.06	25.74	11.22	0	0	0	0.12	0	60.86	0	0.28	25.93	3.98	0	0	0	0.14	0	69.68
Lat_cro	0	18.84	0	0	0	0	0	0	0	81.16	0	3.54	0	0	0	0	0	0	0	96.46
Lat_fro	0	6.93	0	0	0	0	0	0	0	93.07	0	2.06	0	0	0	0	0	0	0	97.94
Lat_lat	0	0.92	0.69	18.52	0	0	0	0	72.01	7.84	0	2.28	0.24	26.46	0	0	0	0	65.81	4.16
Lat_sai	0	4.65	0	0	0	0	0	0	0	95.35	0	2.84	0	0	0	0	0	0	0	97.16
Lat_sch	0	29.71	0	0	0	0	0	0	0	70.29	0	14.02	0	0	0	0	0	0	0	85.98
Lat_sem	0	7.7	0	0	0	0	0	52.8	0	41.07	0	8.46	0	0	0	0	0	17.55	0	77.69
